# Design and assessment of a reconfigurable behavioral assistive robot: a pilot study

**DOI:** 10.3389/fnbot.2024.1332721

**Published:** 2024-02-14

**Authors:** Enming Shi, Wenzhuo Zhi, Wanxin Chen, Yuhang Han, Bi Zhang, Xingang Zhao

**Affiliations:** ^1^State Key Laboratory of Robotics, Shenyang Institute of Automation, Chinese Academy of Sciences, Shenyang, China; ^2^Institutes for Robotics and Intelligent Manufacturing, Chinese Academy of Sciences, Shenyang, China; ^3^University of Chinese Academy of Sciences, Beijing, China; ^4^School of Mechanical Engineering and Automation Northeastern University, Northeastern University, Shenyang, China

**Keywords:** reconfigurable robots, exoskeleton, wearable robots, multi-function, human-robot interaction

## Abstract

**Introduction:**

For patients with functional motor disorders of the lower limbs due to brain damage or accidental injury, restoring the ability to stand and walk plays an important role in clinical rehabilitation. Lower limb exoskeleton robots generally require patients to convert themselves to a standing position for use, while being a wearable device with limited movement distance.

**Methods:**

This paper proposes a reconfigurable behavioral assistive robot that integrates the functions of an exoskeleton robot and an assistive standing wheelchair through a novel mechanism. The new mechanism is based on a four-bar linkage, and through simple and stable conformal transformations, the robot can switch between exoskeleton state, sit-to-stand support state, and wheelchair state. This enables the robot to achieve the functions of assisted walking, assisted standing up, supported standing and wheelchair mobility, respectively, thereby meeting the daily activity needs of sit-to-stand transitions and gait training. The configuration transformation module controls seamless switching between different configurations through an industrial computer. Experimental protocols have been developed for wearable testing of robotic prototypes not only for healthy subjects but also for simulated hemiplegic patients.

**Results:**

The experimental results indicate that the gait tracking effect during robot-assisted walking is satisfactory, and there are no sudden speed changes during the assisted standing up process, providing smooth support to the wearer. Meanwhile, the activation of the main force-generating muscles of the legs and the plantar pressure decreases significantly in healthy subjects and simulated hemiplegic patients wearing the robot for assisted walking and assisted standing-up compared to the situation when the robot is not worn.

**Discussion:**

These experimental findings demonstrate that the reconfigurable behavioral assistive robot prototype of this study is effective, reducing the muscular burden on the wearer during walking and standing up, and provide effective support for the subject's body. The experimental results objectively and comprehensively showcase the effectiveness and potential of the reconfigurable behavioral assistive robot in the realms of behavioral assistance and rehabilitation training.

## 1 Introduction

With the increasing aging of the society's population, the number of individuals with disabilities caused by conditions such as brain injuries and spinal cord injuries is also on the rise (Feigin et al., 2018; Tsao et al., 2022). For patients experiencing lower limb functional movement disorders due to brain or accidental injuries, the restoration of standing and walking abilities plays an important role in clinical rehabilitation. Currently, lower limb rehabilitation robots have garnered significant attention both domestically and internationally (Poggensee and Collins, 2021). Among them, wearable lower limb exoskeleton robots have emerged as one of the most extensively researched solutions, primarily focused on assisting walking and facilitating rehabilitation training (Shi et al., 2021). These exoskeleton robots gather information about the user's intentions by employing electromyography (EMG) sensors to collect EMG signals (Liu et al., 2022; Lu et al., 2022; Li et al., 2023), posture sensors to measure body position (Zeilig et al., 2012; Gao et al., 2020; Bijalwan et al., 2021; Zhang X. X. et al., 2022), and wearable visual odometers (Luo et al., 2022) to determine the body's center of gravity and center of pressure, enabling system control and walking rehabilitation training. However, wearable lower limb exoskeleton robots demand a high degree of balance and locomotor ability from the patients themselves and do not cater to their needs beyond walking in daily activities.

Another critical aspect of daily mobility, apart from walking, is Sit-To-Stand (STS), the ability to transition from a seated to a standing position while maintaining body balance. This task is considerably more demanding than walking, especially with age, as it necessitates coordinated movements of the trunk and lower limbs, as well as muscle strength and control of the center of gravity (COM) in the lower limbs for stability (Galli et al., 2008). Patients with lower limb motor dysfunction often struggle with STS and require additional assistance. For a significant portion of the population, STS poses a significant clinical challenge, impacting their independence in daily life and increasing the risk of falls and injuries. Therefore, from the perspective of addressing users' daily needs, it is imperative for lower limb rehabilitation robots to provide assistance with the standing-up process.

Several research teams have developed assistive robots capable of assisting individuals in transitioning between sitting and standing positions (Fattah et al., 2006; Dall and Kerr, 2010). Several research teams have developed assistive robots capable of assisting individuals to transition between sitting and standing postures (Fattah et al., 2006; Dall and Kerr, 2010). However, their assistive functions are relatively homogenous. Therefore, to enable stable walking for patients after assisted standing and meet their daily activity requirements, some studies have attempted to integrate assistive standing devices with walking assistance devices (Chugo et al., 2007; Asker and Assal, 2019; Huang et al., 2021; Mahdi et al., 2022). These devices help patients transition between sitting and standing positions while providing body support during walking training, enhancing stability, and preventing balance loss (Bell et al., 2021). However, they only provide support during walking, which still relies heavily on the body's own force generation as well as wheel support. This fails to offer sufficient human-computer interaction for gait planning and passive rehabilitation training. Additionally, the integration of wheelchair functions with lower limb rehabilitation robots has not been adequately explored in the process of functional integration.

Assisted standing wheelchairs presently face limitations in delivering walking assistance once the user is in a standing position. Conversely, lower limb exoskeleton robots encounter challenges associated with the sustained maintenance of prolonged and long-distance movements, as well as the necessity for high patient balance. Therefore, in order to meet the needs of patients for a wider range of daily activities, some research teams have proposed wearable and integrated lower limb behavioral assistive robots that can transport users, support standing, and assist with walking in a multifunctional manner (Kong and Jeon, 2005; Hwang and Jeon, 2012; Shankar and Dwivedy, 2015; Li et al., 2019). These robots can transition between wheelchair and exoskeleton modes through disassembly or mechanical transformation to provide versatile assistance. However, they often have drawbacks such as excessive size and weight, tendency to tip over during mode transitions, and inability to support the user in a standing position. In addition, they all have limited effect in passive walking gait training and are unable to perform walking rehabilitation accordingly to the user's needs. These limitations highlight research challenges related to human-machine integration and intelligence in this field.

In this paper, we developed a reconfigurable behavioral assistive robot that can switch between exoskeleton, support, and wheelchair modes through mechanical deformation and reconfiguration to address these issues. First, in the structural design phase, we incorporated a configuration transformation module in the central sagittal plane of the leg, informed by biomechanical data on human walking and sitting processes, to enhance the assisted sitting-up function. Second, we conducted system hardware integration for the reconfigurable behavioral assistive robot, enabling centralized control of all functional modules through the main controller. Additionally, we conducted theoretical kinematic and dynamic analyses of the support state process of the reconfigurable behavioral assistive robot. Finally, we experimentally validated the robot's effectiveness on three levels: gait tracking, EMG signal analysis, and plantar pressure analysis.

The main contributions of this article include: (1) The design of a reconfigurable behavioral assistive robot with a novel mechanism that provides multifunctional assistance for assisted walking, supported standing, assisted standing-up, and wheeled mobility without the need for disassembly. The novel mechanism is based on a four-bar linkage, characterized by a simple switching mechanism and stable reliability, while ensuring a good fit between the robot and the user. (2) The seamless transition between different configurations of the robot and multifunctional fusion control were demonstrated. Experimental validation was conducted not only with healthy subjects but also with simulated hemiparetic patients, confirming the effectiveness of the robot system platform for participants. This presents potential applications for clinical rehabilitation.

To the best of our knowledge, this reconfigurable behavioral assistive robot may be the first one to date that is highly integrated and capable of seamlessly assisting walking, assisting sit-to-stand, supporting standing, and providing wheeled mobility without disassembly. What's more, it has been shown to be effective in both healthy subjects and simulated hemiplegic patients. It addresses the issue of the independence of lower limb rehabilitation equipment and wheeled mobility equipment in existing rehabilitation training modes.

The remainder of this article is organized as follows. Section 2 analyzes the human walking and sitting processes, provides a mechanical structure design, and presents an overview of the working principles of the reconfigurable behavioral assistive robot. This is followed by finite element analysis, system hardware integration, and kinematic and dynamic analysis. Section 3 conducts relevant experiments to validate the effectiveness of the prototype platform of the reconfigurable behavioral assistive robot and presents the experimental results. Section 4 discusses the experimental results, highlights the advantages and disadvantages of the reconfigurable behavioral assistive robot, and outlines future research directions in this field.

## 2 Materials and methods

### 2.1 Analysis of human movement mechanism

#### 2.1.1 Analysis of human walking gait

Walking with a normal gait poses a significant challenge for individuals with lower extremity motor dysfunction. Conducting a gait analysis using Computerized Gait Analysis (CGA) can yield kinematic and kinetic parameters that closely resemble those of a typical human walking gait (Jin et al., 2017). This analysis serves as a valuable reference for the design of the drive system within the exoskeleton module of the reconfigurable behavioral assistive robot. Table 1 presents a summary of the range of motion, the necessary joint torque, and the required joint power for motion in the hip, knee, and ankle joints of the reconfigurable behavior-assisted robot, obtained through CGA gait analysis (Kadaba et al., 1990; Bovi et al., 2011). Furthermore, we have listed the joint motion ranges of the variable reconfigurable behavior-assisted robot in this study, aligning them with the established ranges. These theoretical data serve as a foundation for designing the exoskeleton states for reconfigurable behavior-assisted robots. Consequently, the exoskeleton state of the robot can achieve optimal performance metrics.

**Table 1:** Range of motion, range of joint torque required for motion, and range of joint power required for motion of the human hip, knee, and ankle joints obtained from CGA gait analysis.

**Joint**	**Range of motion (°)**	**Range of motion of robot joints (°)**	**Joint torque range (Nm/kg)**	**Joint power range (W/kg)**
Hip	−10 to 43	−30 to 90	−0.67 to 0.41	−0.70 to 0.73
Knee	0 to 67	0 to 80	−0.15 to 0.43	−0.55 to 0.67
Ankle	−20 to 15	−25 to 25	−0.05 to 1.4	0 to 3

#### 2.1.2 Analysis of human standing-up process

Qian et al. (2006) defined the process of sit-to-stand in normal humans as four stages: the initial preparation stage, the forward leaning storage stage, the forceful standing up stage, and the full standing stage. The third of the four stages is the most critical stage in the process of the human sit-to-stand, and the standing process of patients with lower limb dysfunction is mainly limited by the third stage. Therefore, the human sit-up assistive device mainly supports the human body around this stage. Considering that the arm strength of the target population of the robot is generally low, this study proposes to use knee support to assist patients with lower limb motor dysfunction to complete the sit-to-stand transition, i.e., fixing the ankle joint, supporting the knee joint, and lifting the user by traction on the trunk.

**Figure 1:**
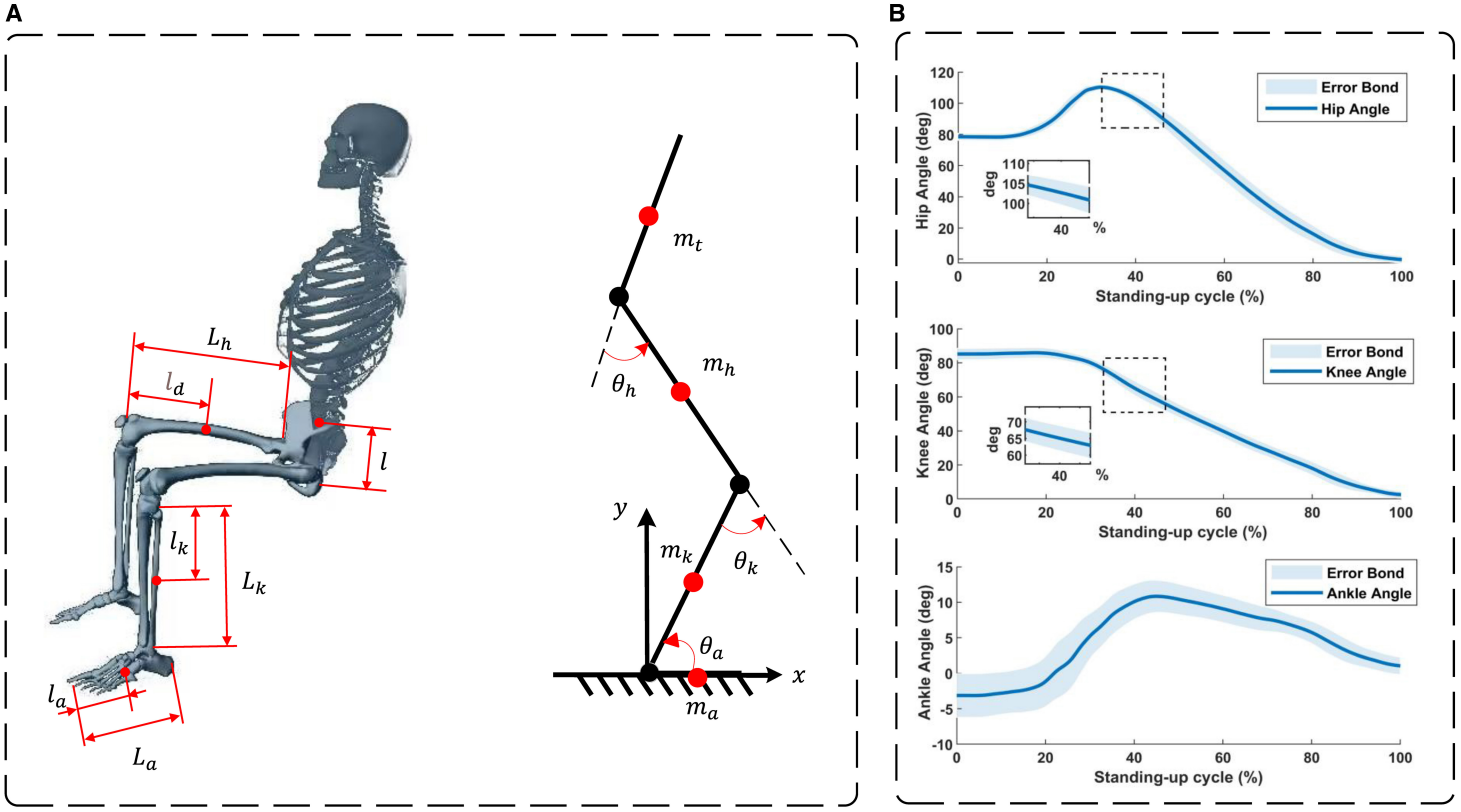
**(A)** A STS model of the human body. **(B)** Hip, knee, and ankle joint angles during sit-to-stand motion in 20 healthy subjects.

Motion analysis serves as a powerful tool for quantitative assessment of human mobility, including body kinematics and dynamics analysis. Its applications extend to structural design, the evaluation of treatment programs, monitoring of human abnormalities, and more. Vector analysis is used to solve the kinematics of the human body in terms of positive and inverse solutions and to analyze the kinematic properties of the lower limbs using the Lagrange method. The human body is considered as a multibody dynamics model with three degrees of freedom consisting of the trunk, hip, knee, ankle and foot, as shown in Figure 1A. The masses of the trunk, thigh, calf, and foot are *m*_*t*_, *m*_*h*_, *m*_*k*_, and *m*_*a*_, respectively; the lengths of the thigh, calf, and foot are *l*_*h*_, *l*_*k*_, and *l*_*a*_, respectively; the distances between the centers of mass and the joints of the trunk, thigh, calf, and foot are *l*, *l*_*h*_, *l*_*k*_, and *l*_*a*_, respectively; and the joint angles of the hip, knee, and ankle are θ_*h*_, θ_*k*_, and θ_*a*_, respectively.

According to the STS model, the mass center coordinates of foot are


(1) $$\begin{array}{l} h_{ax}\;=\;l_{a},\;h_{ay}=0 \end{array}$$

the mass center coordinates of shank are


(2) $$\begin{array}{l} h_{kx}\;=\;\left(L_{k}-l_{k}\right)\cos\theta_{a};\;h_{ky}\;=\;\left(L_{k}-l_{k}\right)\sin\theta_{a} \end{array}$$

the mass center coordinates of thigh are


(3) $$\{\begin{array}{c} h_{hx}\;=\;L_{k}\sin\theta_{a}-l_{h}\cos(180-\theta_{a}-\theta_{k})\\ h_{hy}\;=\;L_{k}\cos\theta_{a}+l_{h}\sin(180-\theta_{a}-\theta_{k}) \end{array}$$

the mass center coordinates of trunk are


(4) $$\{\begin{array}{c} h_{tx}\;=\;L_{k}\sin\theta_{a}-l_{h}\cos\left(180-\theta_{a}-\theta_{k}\right)\;+\;l\cos(\theta_{k}\;+\theta_{a}-\theta_{h})\\ h_{ty}\;=\;L_{k}\cos\theta_{a}\;+l_{h}\sin(180-\theta_{a}-\theta_{k})\;+\;l\sin(\theta_{k}+\theta_{a}-\theta_{h}) \end{array}$$

Taking the derivatives of Equations (2–4) with respect to time provides the velocities of the center of mass for the torso, thigh, and shin, respectively. Subsequently, the total kinetic energy of a single leg is obtained.


(5) $$\begin{array}{l} T=\frac{1}{2}m_{k}v_{k}{\;}^{2}+\frac{1}{2}J_{C_{k}}{\dot{\theta }}_{a}^{2}+\frac{1}{2}m_{h}v_{h}{\;}^{2}+\frac{1}{2}J_{C_{h}}{\dot{\theta }}_{k}^{2}+\frac{1}{2}m_{t}v_{t}{\;}^{2}+\frac{1}{2}J_{C_{t}}{\dot{\theta }}_{h}^{2}\\ \;=\frac{1}{2}m_{k}{\left[(L_{k}-l_{k}){\dot{\theta }}_{a}\right]}^{2}+\frac{1}{2}J_{C_{k}}{\dot{\theta }}_{a}^{2}\\ \;+\frac{1}{2}m_{h}\left[L_{k}{\;}^{2}+l_{h}{\;}^{2}+2L_{k}l_{h}\sin\left(\theta_{a}-\hat{\theta }\right){\dot{\theta }}_{a}^{2}+l_{h}{\;}^{2}{\dot{\theta }}_{k}^{2}\right]+\frac{1}{2}J_{C_{h}}{\dot{\theta }}_{k}^{2}\\ \;+\frac{1}{2}m_{t}\left[L_{k}{\;}^{2}+l_{h}{\;}^{2}+l^{2}+2L_{k}l_{h}\sin\left(\theta_{a}-\hat{\theta }\right)\;\right]{\dot{\theta }}_{a}^{2}{\dot{\theta }}_{h}^{2}\\ \;-m_{t}\left[L_{k}l\sin\left(\theta_{a}+\bar{\theta }\right)+L_{h}l\cos\left(\hat{\theta }+\bar{\theta }\right)\;\right]{\dot{\theta }}_{a}^{2}{\dot{\theta }}_{h}^{2}\\ \;+\frac{1}{2}m_{t}\left[L_{h}{\;}^{2}+l^{2}-2L_{h}l\cos\left(\hat{\theta }+\bar{\theta }\right)\right]{\dot{\theta }}_{k}^{2}l^{2}{\dot{\theta }}_{h}^{2}+\frac{1}{2}J_{C_{t}}{\dot{\theta }}_{h}^{2} \end{array}$$

where $\hat{\theta }=180-\theta_{a}-\theta_{k}\;,\;\bar{\theta }=\theta_{k}+\;\theta_{a}-\theta_{h}$

The total potential energy of single leg is *P*


(6) $$\begin{array}{r} P\;=\;m_{k}g\left(L_{k}-l_{k}\right)\sin\theta_{a}+m_{h}g\left(L_{k}\cos\theta_{a}+l_{h}\sin\left(180-\theta_{a}-\theta_{k}\right)\right)\;\\ \;+m_{t}g\left(L_{k}\cos\theta_{a}+L_{h}\sin\left(180-\theta_{a}-\theta_{k}\right)\right)\;\\ +l\sin\left(\theta_{k}+\theta_{a}-\theta_{h}\right)\; \end{array}$$

The Lagrange equation is defined as the difference between kinetic energy (*T*) and potential energy (*P*). Therefore, from Equations (5) and (6), the Lagrange equation and the joint moment are obtained as shown in Equations (7) and (8).


(7) $$\begin{array}{l} L=T-P \end{array}$$

(8) $$\begin{array}{l} M_{j}\;=\;\frac{d}{dt}\frac{\partial L}{\partial \dot{\theta_{j}}}-\frac{\partial L}{\partial \theta_{j}} \end{array}$$

where $\theta_{j},\;\dot{\theta_{j}}$ are the generalized angle and generalized velocity of the hip, knee, and ankle, respectively.


(9) $$\begin{array}{l} P_{i}\;=\;M_{i}{\dot{\theta }}_{j} \end{array}$$

The joint power of the human body are described as the working capacity of the joints; according to Equation (9), the power of the joints of the lower limbs is obtained, respectively. It provides a theoretical basis and data support for the structural design of the robot as well as the selection of drive elements.

In order to ensure precise module alignment during the structural design phase of the reconfigurable behavioral assistive robot, while also achieving a consistent motion trajectory, we conducted data collection from 20 healthy subjects who had no history of disability. These individuals were observed during the sit-to-stand transition while utilizing knee support. Data acquisition was facilitated through the use of a wearable motion capture system (200 Hz; myoMOTION, Noraxon USA Inc.), attached to both the bilateral thigh, shank, and foot regions. Subsequently, the collected data underwent preprocessing using MR software (myoRESEARCH, Noraxon USA Inc.). The resulting hip, knee, and ankle joint angle variations, along with their associated error bands, are depicted in Figure 1B. These angle measurements offer a robust theoretical and empirical foundation for guiding the structural design of the reconfigurable behavioral assistive robot.

### 2.2 Design of the reconfigurable behavioral assistive robot

#### 2.2.1 Structural design strategy

**Figure 2:**
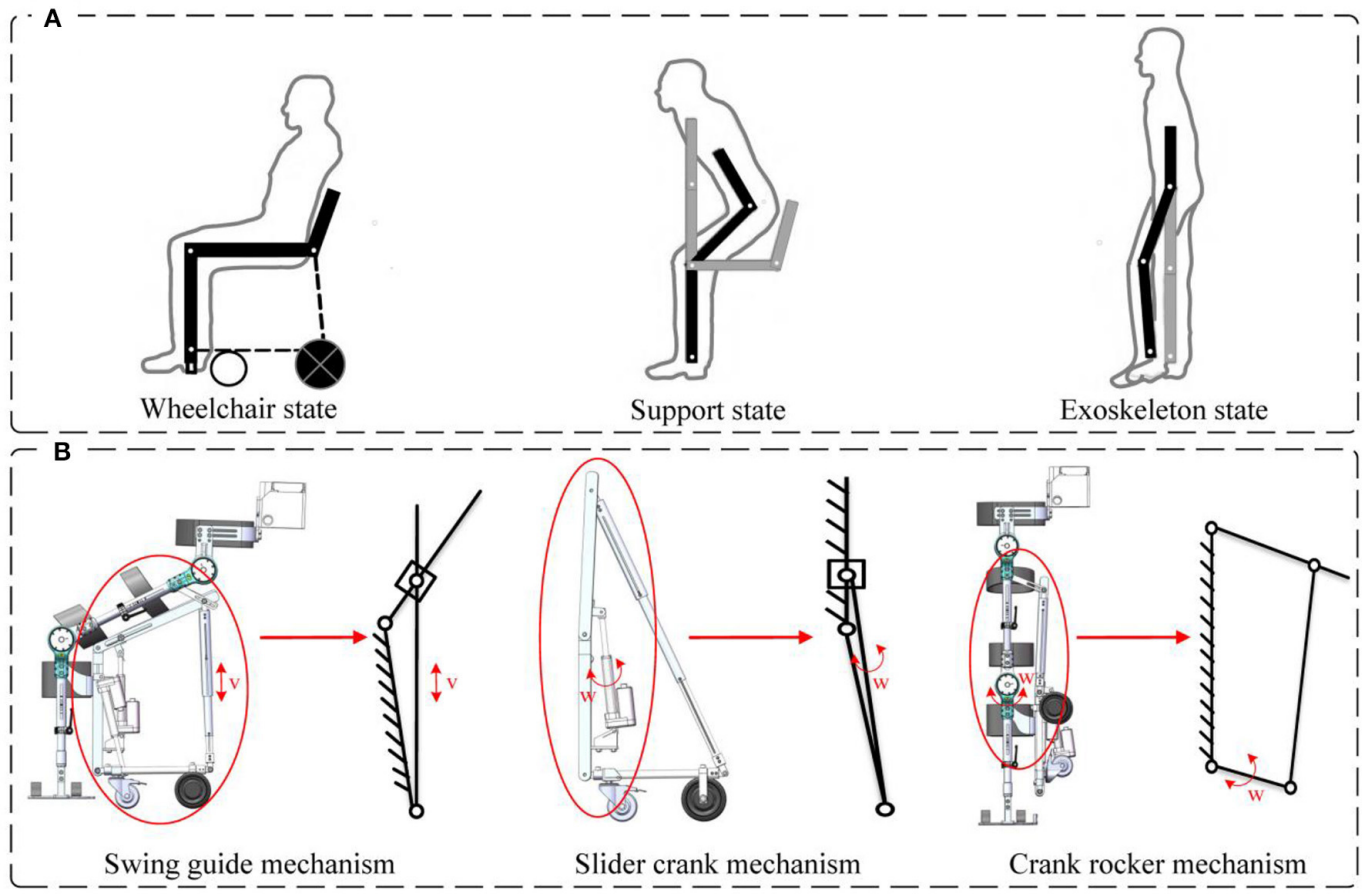
**(A)** Simplified modeling of three configurations of the robot. **(B)** Schematic diagram of the design of the conformal transformation module.

The reconfigurable behavioral assistive robot aims to aid individuals with lower extremity motor dysfunction in their daily activities, including walking, sitting, and wheelchair use. As such, the robot's configuration strategy must ensure its adaptability to three key states: wheelchair, support, and exoskeleton states. Simplified models of these configurations are depicted in Figure 2A. Additionally, the robot should seamlessly and reliably transition between these three states via a reconfigurable mechanism, enabling functions such as robot-assisted walking, assisted standing-up, and wheelchair mobility. In the exoskeleton state, the robot's structure should closely mimic the physiological structure of the human lower limb. Specifically, it should provide two active degrees of freedom for the hip and knee joints and passive degrees of freedom for the ankle joint. Each component should be adjustable to accommodate variations in lower limb bone length among different users, and the joint angles should align with the normal range of motion of human lower limb joints during walking. For the support state, the robot employs a knee support and ankle joint fixation approach. Furthermore, the conformal transformation module must offer sufficient support to facilitate the user's ability to stand up and maintain stability throughout the standing-up process, regardless of the chosen support angle. In the robotic wheelchair state, the conformal transformation module lacks degrees of freedom, except for the wheels. The user should be able to sit comfortably and securely on this configuration change module. Based on the above design principles, the configuration scheme for the exoskeleton module of the variable conformation exoskeleton robot and the conformal transformation module is determined as follows:

(1) Exoskeleton module: the exoskeleton module considers only sagittal plane movement of the lower limbs. It provides one degree of freedom for hip and knee joint flexion/extension, with the option to include a passive degree of freedom for top flexion/dorsiflexion at the ankle joint for enhanced versatility. The exoskeleton state's configuration design should be adjustable to accommodate varying human body sizes and fit users within the height range of 157–181 cm. Adjustable range of thigh linkage is 410–520 mm, calf linkage is 330–420 mm and waist width is 270–360 mm.
(2) Conformal transformation module: to optimize the assisted sitting function and ensure wheelchair comfort, an innovative conformal transformation module is positioned along the central sagittal plane of the leg. The articulation centers for wheel linkage and telescopic rods align with the rotation centers of the thigh and calf support rods to synchronize with the knee joint movement of the exoskeleton module. This is achieved through a four-bar mechanism, introducing an additional degree of freedom. In the supported state, the thigh and calf support mechanism functions as a swing guide mechanism. In the exoskeleton state, the thigh support mechanism adopts a slider-crank mechanism, while the calf support rod can be affixed to the exoskeleton module's calf linkage. The thigh support rod and the exoskeleton module's thigh linkage form a crank-rocker mechanism. Furthermore, a mobility sub-component is incorporated into the thigh support articulation to ensure a comfortable fit for users with the conformal transformation module in both supported and wheelchair states, without disrupting the exoskeleton's gait trajectory. A schematic diagram of the conformal transformation module's design is presented in Figure 2B.

#### 2.2.2 Overall mechanical structure

**Figure 3:**
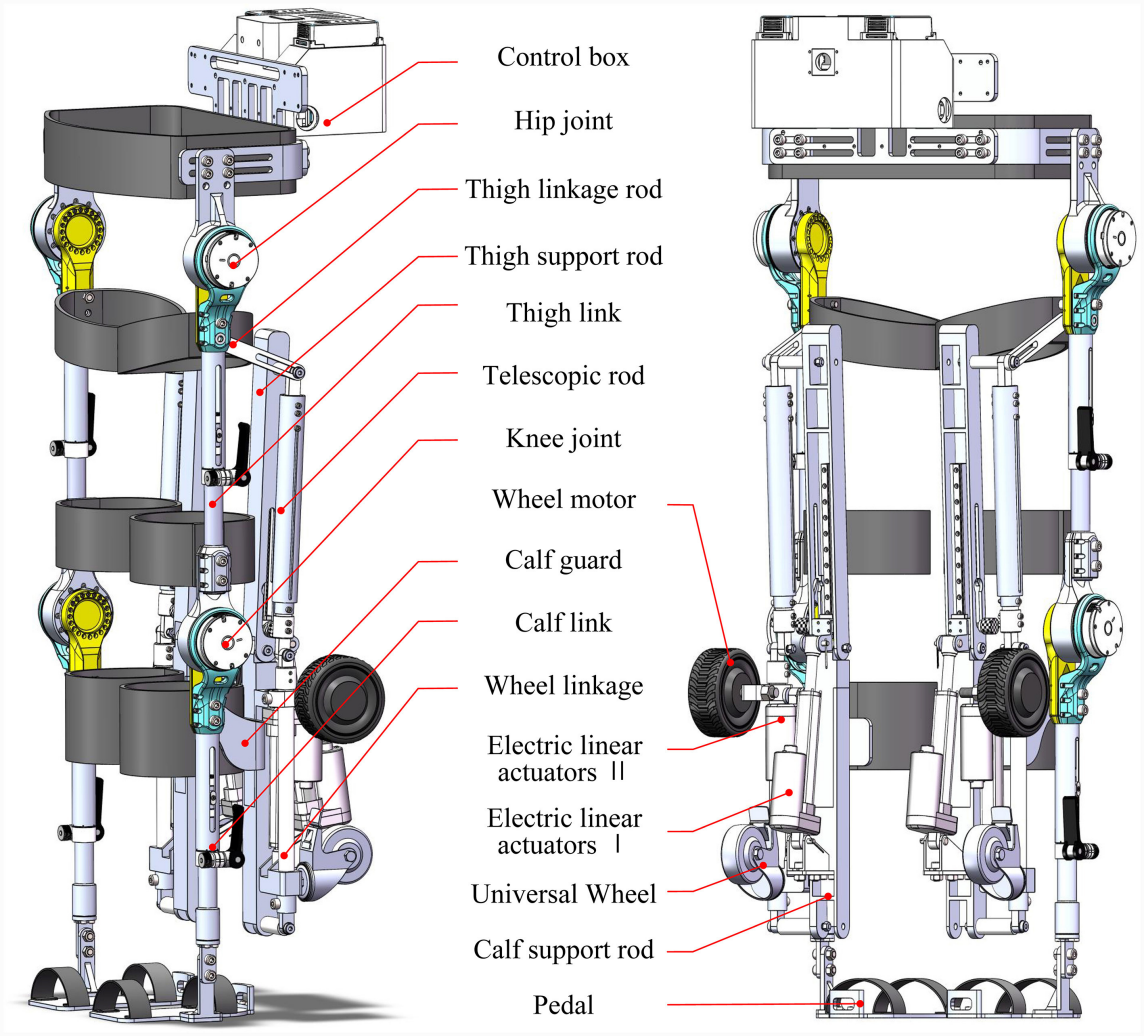
Overall structure of the reconfigurable behavioral assistive robot.

The comprehensive structure of the reconfigurable behavioral assistive robot is depicted in Figure 3, comprising both the exoskeleton module and the conformal transformation module. Notably, the calf support rod of the conformal transformation module can be threaded onto the calf link of the exoskeleton module. The thigh support rod and the thigh link of the exoskeleton module collaborate to create a crank rocker mechanism, incorporating an opening slot in the link to ensure a snug fit between the user and the conformal transformation module. For the joint drive motor of the exoskeleton module, the QDD Pro-PR60-100-80 intelligent integrated joint is selected. Meanwhile, the conformal transformation module utilizes electric linear actuators commonly found in rehabilitation machinery. In this study, aluminum alloy serves as the primary material for the main body of the reconfigurable behavioral assistive robot, with mild steel employed for components necessitating higher strength.

**Figure 4:**
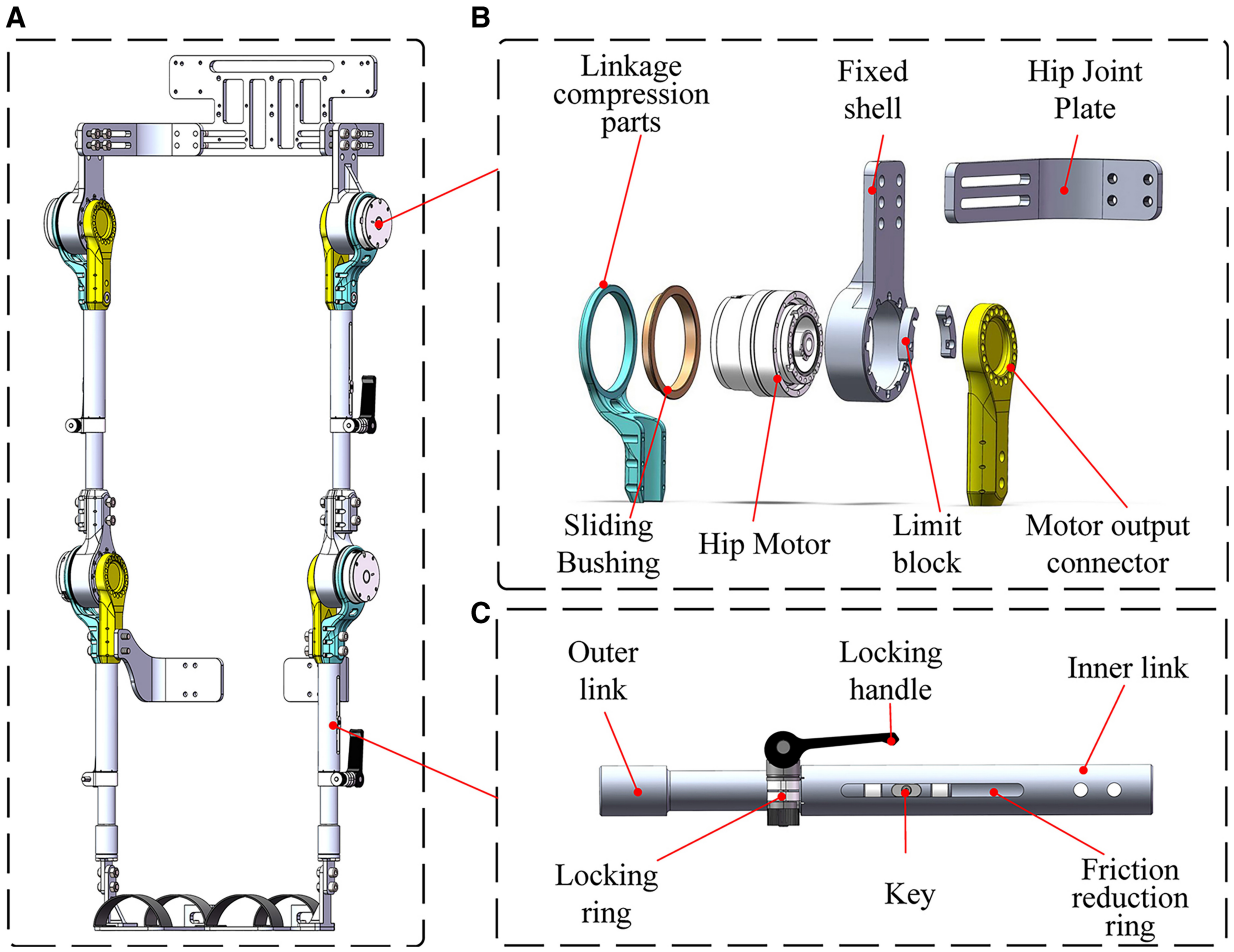
**(A)** Overall structure of the exoskeleton module. **(B)** Mechanical structure of the hip joint. **(C)** Mechanical structure of the calf link adjustable device.

The exoskeleton module encompasses the waist, control box, and symmetrical left and right hip, knee, ankle, thigh links, calf links, and foot pedals, as illustrated in Figure 4A. The hip joint comprises the hip motor, hip fixation shell, and hip flexion and extension components, with mechanical limitations imposed on hip joint motion in the sagittal plane to ensure user safety. Both left and right sides of the hip joint exhibit symmetry, with the mechanical structure of the right portion displayed in Figure 4B. Similarly, the knee joint is composed of the knee motor, knee fixation housing, and knee flexion and extension assembly, featuring mechanical restrictions on knee joint motion in the sagittal plane. The knee joint is symmetrical on both sides and bears mechanical similarities to the hip joint. To accommodate users of various heights and sizes, the leg link length of the reconfigurable behavioral assistive robot is adjustable. The design principles of the thigh link and calf link adjustable device are identical. Taking the calf rod adjustable device as an example, its structure encompasses the outer link, inner link, locking handle, locking ring, key, and friction-reducing ring, as depicted in Figure 4C. The waist's width and the hip's front-to-back distance can be finely adjusted by altering the relative fastening positions of screws, thereby catering to individuals with different waist circumferences.

**Figure 5:**
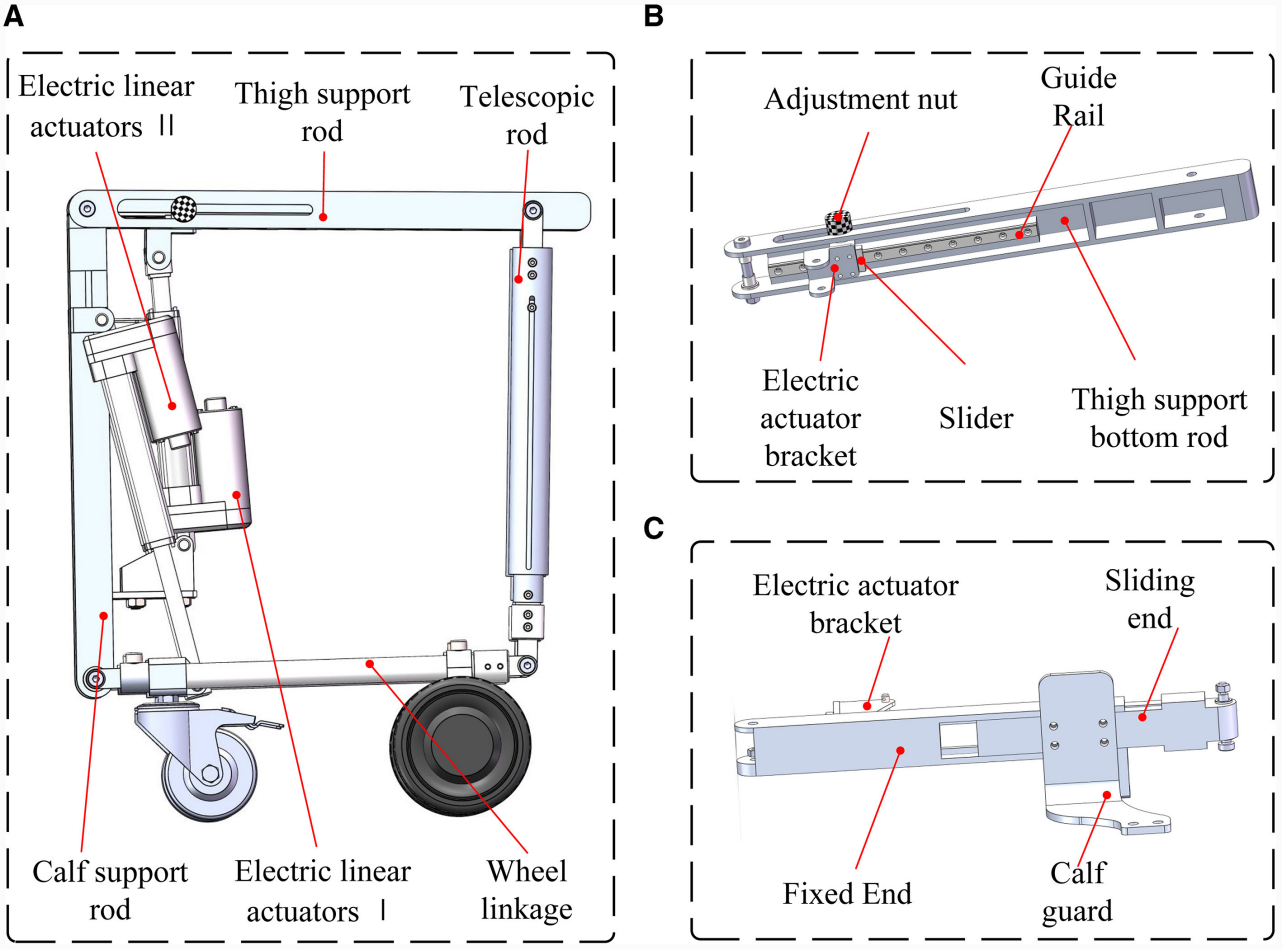
**(A)** Overall structure of the conformal transformation module. **(B)** Mechanical structure of the thigh support rod. **(C)** Mechanical structure of the calf support rod.

The conformal transformation module comprises the thigh support rod, calf support rod, wheel linkage, telescopic bar, electric linear actuator 1, electric linear actuator 2, universal wheel, and wheel motor, as showcased in Figure 5A. The thigh support rod offers a fitting support surface for the user, including a thigh support bottom rod, slider guide, slider, screw, adjustment nut, and push rod holder, as seen in Figure 5B. Adjusting the depth of the adjustment nut securely fixes and limits the slider, enabling configuration changes. The calf support rod's structure, shown in Figure 5C, accommodates two electric linear actuators to provide support in both the support and wheelchair states. This component is divided into two sections and features a slot to align with the slide. During the transition to a wheelchair state, the electric linear actuator extends upward to a specified length. The pusher then elevates the thigh support rod and the sliding end of the calf support rod to a certain height, lifting the robot's feet off the ground and enabling smooth wheeled movement. Additionally, the sliding end of the calf support rod is equipped with screw adjustment holes, facilitating the adjustment of the calf guard's relative position on the calf support rod to accommodate users of varying heights and body types while wearing the exoskeleton module.

#### 2.2.3 Principle of conformal transformation

The transition between the robot's three configurations must ensure both reliable and stable switching and the wearer's comfort, particularly in terms of the hip leaning surface, as well as disengaging the foot pedals from the ground during wheelchair travel. In light of these requirements, this paper introduces an innovative scheme for transforming the wheelchair state configuration, employing a four-bar mechanism as the fundamental configuration when switching between the conformal transformation module's wheelchair state and support state, as illustrated in process A. This transformation employs two linear actuators for operation. Linear actuator 1 propels the sliding rod to rotate the seat plate around the knee joint, assisting the wearer in transitioning from a seated position to standing by following the motion of the thigh support bar. Meanwhile, linear actuator 2 supports the wheel bar. The calf support rod is equipped with a mobile vice, allowing it to move when transitioning from a supported state to a seated position in the wheelchair state. Linear actuator 1 is reactivated, sliding the upper end of the calf support rod upward and taking the wearer's calf along, thereby lifting the plantar pedal off the ground.

**Figure 6:**
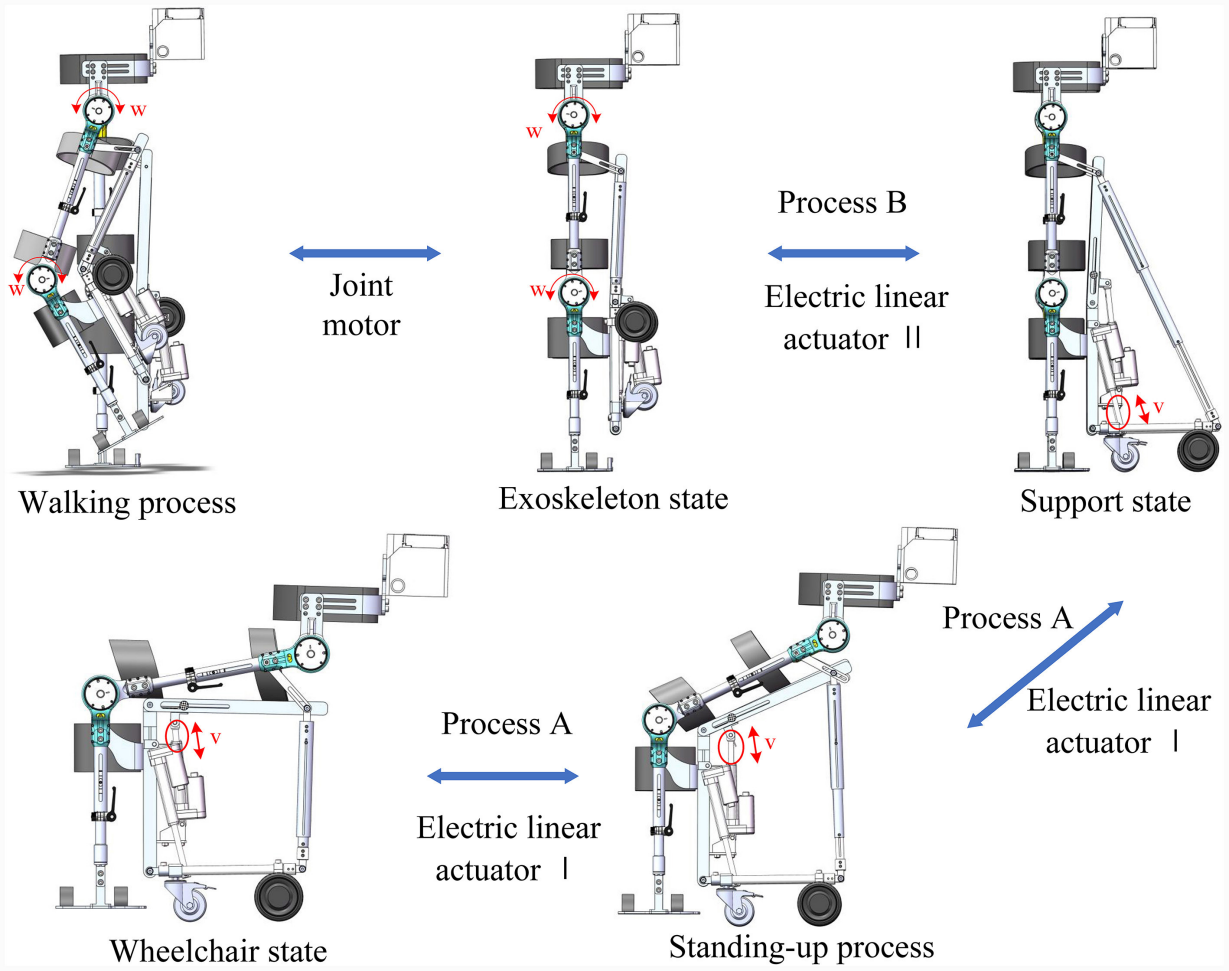
Schematic diagram of the conformational transformation process.

When shifting between the robot's support state and exoskeleton state, as depicted in process B, linear actuator 1 elevates the wearer to a standing position by driving the thigh support rod. Subsequently, after standing, linear actuator 2 contracts to retract the wheel linkage, causing the telescopic rod to contract. This results in the wheel linkage and the hinge center of the calf support rod becoming coaxial. Simultaneously, the thigh support mechanism transforms into a swing guide mechanism with one degree of freedom. The schematic diagram of this conformal transformation is presented in Figure 6.

#### 2.2.4 Finite element analysis

In this section, we utilized the Ansys Workbench platform to conduct finite element static analysis on both the exoskeleton module in four typical gait states and the sit-to-stand transition process within the conformal transformation module.

Following the establishment of constraints and the application of loads, the safety factors obtained from the analysis and calculations performed on the Ansys Workbench platform indicated that all safety factors for the exoskeleton module during the four gait phases exceeded 1. Similarly, the safety factor obtained from analyzing the four key support phases during the sit-to-stand transition of the conformal transformation module was also greater than 1. Consequently, it can be concluded that the material strength of both the exoskeleton module and the conformal transformation module meets the operational requirements.

#### 2.2.5 System integration

**Figure 7:**
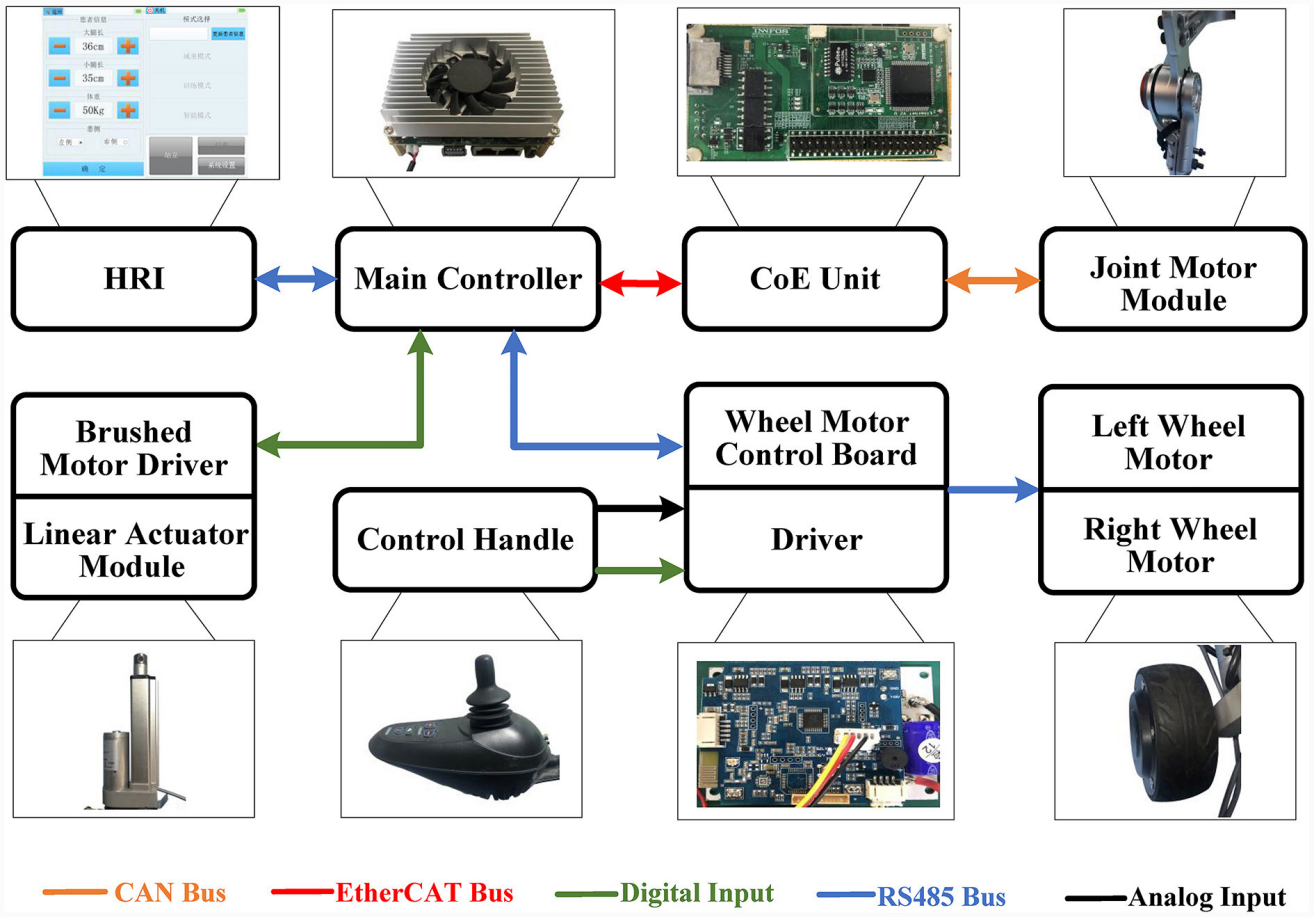
System integration of reconfigurable behavioral assistive robots.

The system integration of the reconfigurable behavioral assistive robot is presented in Figure 7. This diagram illustrates the coordinated operation of various components within the system: the exoskeleton hip motor module and knee motor module (specifically, the QDD Pro-PR60-100-80 units from Mintasca, China) establish data exchange with the main controller (PICO-TGU4, AAEON, China) through the CoE (CAN over EtherCAT) protocol. Simultaneously, the linear actuator (SY-A02B, OURUIDE, China) situated in the configuration transformation module is seamlessly integrated into the main controller. Here, the main controller governs the initiation and termination of the linear actuator motor via digital output signals. Control of the wheel motor (HB-105, Jindouyun, China) is also incorporated into the main controller. Additionally, the control handle inputs both analog and digital signals into the hub motor control board. This board is responsible for executing the start-stop and steering control of the wheel motor via the RS485 protocol. The peak torque of the joint motors in the robot exoskeleton module is 108 Nm, the linear actuator motors in the configuration change module have an extension of 1,000 mm and a thrust force of 1,500 N, and the hub motors of the wheelchair state are selected to be 4-inch single-side-axis hub motors, with an expected maximum traveling speed of 7 km/h. Furthermore, the human-machine interface is integrated into the main controller using the RS485 protocol, establishing a communication link between the user and the system. This comprehensive system integration ensures the smooth and coordinated operation of the reconfigurable behavioral assistive robot's diverse components, contributing to its overall functionality and usability.

### 2.3 Dynamic characteristics analysis

This section concentrates on analyzing the dynamic characteristics of the robot's support state, which serves as the primary focus of the study. In the support state, both electric linear actuators are actively engaged. Given the known feed speed of the electric linear actuators, it becomes possible to calculate the position of the configuration change mechanism through kinematic analysis. Additionally, the inverse dynamics of the support state will be modeled independently. This modeling aims to determine the required driving moment for each click of the linear actuator during the sit-to-stand transition within the support state. This comprehensive analysis provides insights into the dynamic behavior and performance of the robot in its support configuration, contributing to a better understanding of its functionality during critical tasks like the sit-to-stand transition.

#### 2.3.1 Support state kinematic analysis

**Figure 8:**
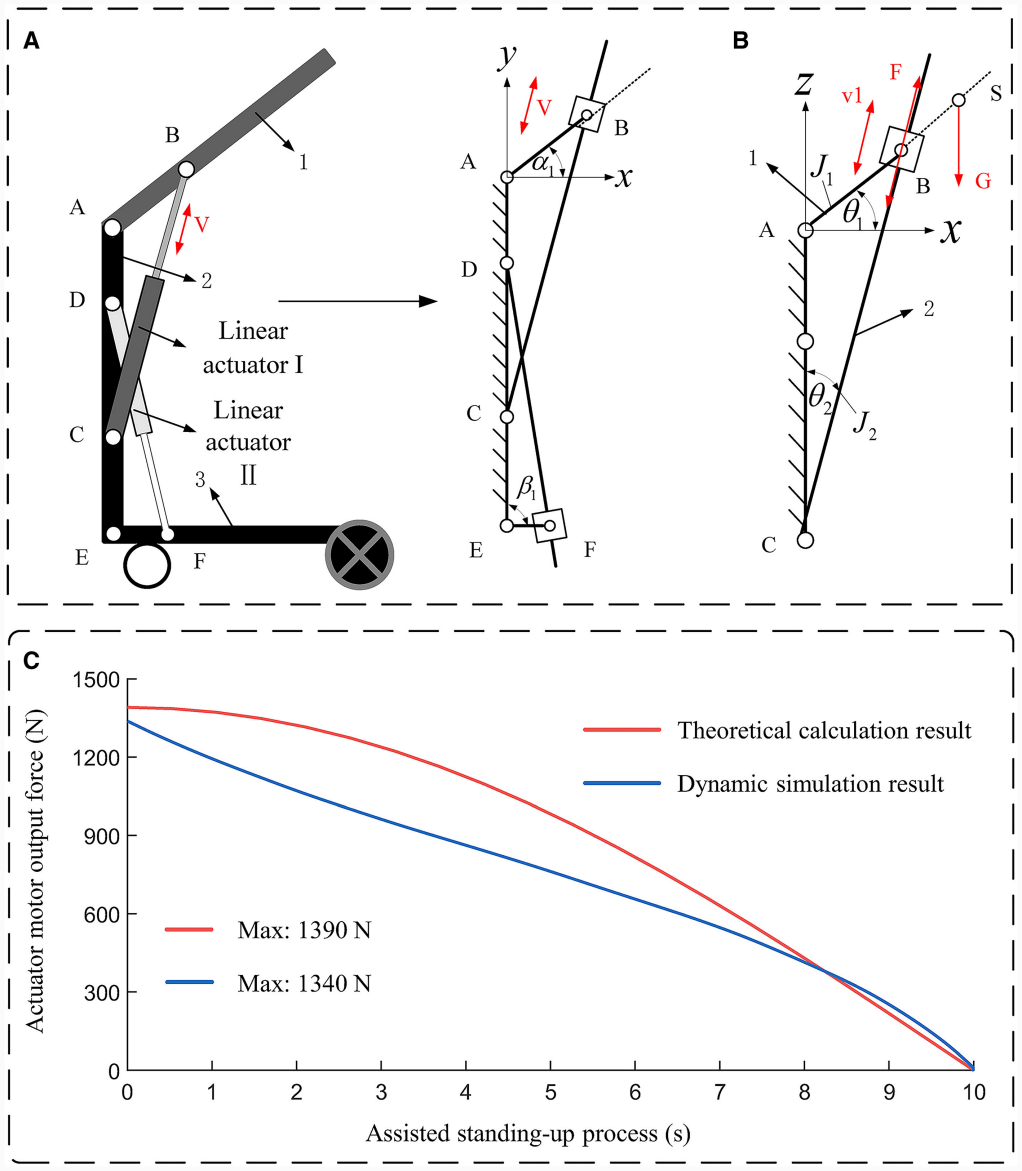
**(A)** Kinematic model of support state. **(B)** Kinetic model of support state. **(C)** Dynamic analysis result.

In the support state, the conformal transformation module aids individuals with lower limb motor dysfunction in transitioning from a sitting to a standing posture. Figure 8A provides an illustration of the mechanism involved in this process. During the conversion from sitting to standing, connecting rod 1 and 2, along with connecting rod 2 and 3, together form a swing guide mechanism with electric linear actuators I and II, respectively. Electric linear actuator I drives connecting rod 1 to rotate around the knee joint, while electric linear actuator II retracts connecting rod 3. In this context, we analyze the changes in angular displacement θ and angular velocity *w* of the connecting rod under the influence of the electric linear actuators based on the geometric relationships of motion.

For electric linear actuator I, the relationship between the rotation angle α_1_ of connecting rod 1 and the feed amount *s*_1_ of electric linear actuator I is obtained from the cosine theorem as follows:


(10) $$\begin{array}{l} \alpha_{1}\;=\;\sin^{-1}\left(\frac{{l_{AB}}^{2}+{l_{AC}}^{2}-{\left(l_{BC}+s_{1}\right)}^{2}}{2l_{AB}l_{AC}}\right) \end{array}$$

where, *l*_*AB*_ indicates the distance between the center of rotation of connecting rod 1 and the hinge center of electric linear actuator I, *l*_*AC*_ indicates the distance between the center of rotation of connecting rod 1 and the hinge center of electric linear actuator II, and *l*_*BC*_ indicates the length of *l*_*AB*_ in the sitting position, i.e., the initial length of electric linear actuator I. The relationship between the angular velocity *w*_1_ of the connecting rod 1 during the support process and the feed amount *s*_1_ and feed speed *v*_1_ of the electric linear actuator I is obtained by deriving both sides of Equation (10), as shown in Equation (11)


(11) $$\begin{array}{l} w_{1}\;=\;\frac{-2\left(l_{BC}+s_{1}\right)}{\sqrt{4{l_{AB}}^{2}{l_{AC}}^{2}-{\left[{l_{AB}}^{2}+{l_{AC}}^{2}-{\left(l_{BC}+s_{1}\right)}^{2}\right]}^{2}}}v_{1} \end{array}$$

For electric linear actuator II, the relationship between the rotation angles β_1_ and *w*_2_ of connecting rod 3 and the feeds *s*_2_ and *v*_2_ of electric linear actuator I is obtained from the cosine theorem:


(12) $$\begin{array}{l} \beta_{1}\;=\;\arccos\left(\frac{{l_{DE}}^{2}+{l_{EF}}^{2}-{\left(l_{DF}+s_{2}\right)}^{2}}{2l_{DE}l_{EF}}\right) \end{array}$$

(13) $$\begin{array}{l} w_{2}\;=\;\frac{2\left(l_{DF}+s_{2}\right)}{\sqrt{4{l_{DE}}^{2}{l_{EF}}^{2}-{\left[{l_{DE}}^{2}+{l_{EF}}^{2}-{\left(l_{DF}+s_{2}\right)}^{2}\right]}^{2}}}v_{2} \end{array}$$

in Equations (12, 13), *l*_*DE*_ denotes the distance between the two electric linear actuator hinge centers on connecting rod 2, *l*_*EF*_ denotes the distance between the center of rotation of connecting rod 3 and the hinge center of electric linear actuator II, and *l*_*DF*_ denotes the length of *l*_*DF*_ in the exoskeletal state i.e., the initial length of electric linear actuator II when connecting rods 2 and 3 overlap.

#### 2.3.2 Support state dynamics analysis

When the conformal transformation module is in the support state, connecting rods 1 and 2, along with electric linear actuator I, form the oscillating guide mechanism. Among them, the linear actuator I plays a dominant role in the robot-assisted human sit-to-stand transition. Therefore, in the dynamics analysis of the robot's support state, we focus on the analysis of linear actuator I. According to D'Alembert's principle, this mechanism is considered to be in a state of force equilibrium, taking into account the inertial forces acting on each member as external forces applied to the respective member. Furthermore, due to the complete symmetry of the reconfigurable behavioral assistive robot's left and right sides, the analysis of the driving force required for single-leg support focuses solely on one lower limb. The established inverse dynamics model is illustrated in Figure 8B.

According to the principle of imaginary displacement the following relation can be written:


(14) $$\begin{array}{l} \sum \left(F_{i}ds_{i}+T_{i}d\theta_{i}\right)=0 \end{array}$$

(15) $$\begin{array}{l} \theta_{2}\;=\;\arccos\left(\frac{{l_{AC}}^{2}+{\left(l_{BC}+s_{1}\right)}^{2}-{l_{AB}}^{2}}{2l_{AC}\left(l_{BC}+s_{1}\right)}\right) \end{array}$$

(16) $$\begin{array}{l} T_{1}\;=\;-mgl_{AS}\cos\theta_{1} \end{array}$$

(17) $$\begin{array}{l} T_{2}\;=\;-J_{1}{\ddot{\theta }}_{1} \end{array}$$

(18) $$\begin{array}{l} T_{3}\;=\;-J_{2}{\ddot{\theta }}_{2} \end{array}$$

Dividing the above equation by the differential time *d*_*t*_ and substituting it gives the following equation:


(19) $$\begin{array}{l} F=\frac{\left(mgl_{AS}\cos\theta_{1}{\dot{\theta }}_{1}+J_{1}{\ddot{\theta }}_{1}{\dot{\theta }}_{1}+J_{2}{\ddot{\theta }}_{2}{\dot{\theta }}_{2}\right)}{v_{1}} \end{array}$$

where *F* is the driving force of electric linear actuator I, *m* is the coupling mass of human body and robot thighs and upper body torso, *l*_*AS*_indicates the distance from the center of rotation of connecting rod 1 to the projection of the coupling mass of robot thighs and upper body torso on connecting rod 1, *J*_1_ is the rotational inertia of connecting rod 1, *J*_2_ is the rotational inertia of connecting rod 2.

The conformal transformation module of the reconfigurable behavioral assistive robot needs to drive the human body and the exoskeleton module to move together as a unified whole to assist the subject to complete the sit-to-stand transition. Therefore, it is necessary to incorporate parameters such as coupling quality, center of mass, and moments of inertia for each part of the human body and exoskeleton module into the model. During the sit-to-stand movement, the motion of the conformal transformation module primarily occurs in the sagittal plane. Therefore, the coupling of their centers of mass only considers coupling within the sagittal plane, and the coupling of their moments of inertia only considers coupling along the coronal axis.

In the calculation of center of mass coupling, let the coordinates of the projection of the center of mass *m*_*i*_ of a certain part of the human body in the sagittal plane are (*x*_*hi*_, *y*_*hi*_), and the coordinates of the center of mass *m*_*j*_ of the corresponding part of the exoskeleton module in the sagittal plane are (*x*_*ri*_, *y*_*ri*_), then the coupled center of mass (*x*_*ci*_, *y*_*ci*_) satisfies:


(20) $$\{\begin{array}{c} x_{ci}\;=\;x_{hi}+\frac{m_{ri}(x_{ri}-x_{hi})}{m_{hi}+m_{ri}}\\ y_{ci}\;=\;y_{hi}+\frac{m_{ri}(y_{ri}-y_{hi})}{m_{hi}+m_{ri}} \end{array}$$

The coupled rotational inertia *J*_*hr*_ in the direction of the coronal axis satisfies the parallel axis theorem of rotational inertia. That is:


(21) $$\begin{array}{l} J_{hr}\;=\;J_{hi}+J_{ri}+m_{hi}\left[{\left(x_{ci}-x_{hi}\right)}^{2}+{\left(y_{ci}-y_{hi}\right)}^{2}\right]\\ \;+m_{ri}\left[{\left(x_{ci}-x_{ri}\right)}^{2}+{\left(y_{ci}-y_{ri}\right)}^{2}\right] \end{array}$$

where *J*_*hi*_ and *J*_*ri*_ are the respective moment of inertia of each part of the human body and the corresponding part on the exoskeleton module, respectively.

The mass, center of mass and rotational inertia parameters of the exoskeleton module man-machine coupling calculated by Equations (20, 21) are shown in Table 2.

**Table 2:** The human-robot coupling parameters of the exoskeleton module.

**Human-robot coupling**	**Coupled mass m (kg)**	**Distance from coupled center of mass to joint d (m)**	Coupled rotational inertia J (kg^*^m^2^)
Thigh	17.21	0.226	0.187
Calf	4.74	0.160	0.035
Foot	1.82	(0.106, 0.024)	0.005
Upper trunk	62.74	0.376	0.204

Numerical simulation of the robot support state dynamics model established by Equations (14–21) is performed by MATLAB to calculate the change in the value of the output force of the linear actuator I while the robot is supporting the human body to stand up. Meanwhile, the coupled rotational inertia of the thigh and upper limb trunk in the table is added to the parameters of the thigh support rod in SolidWorks software, and the coupled mass of the thigh and upper limb trunk is added in the form of an external force at the coupled center of mass of the thigh and upper limb trunk, respectively. Conducting dynamic simulation of the robot's support state through the Motion module in SolidWorks software. The output force of linear actuator 1 in the process of standing up is obtained. The results of the theoretical calculations and dynamics simulations are shown in Figure 8C.

As shown in Figure 8C, the maximum output force of linear actuator I calculated by kinetic modeling is 1,390 N in the process of robot-assisted subject sit-to-stand transition, and the maximum output force of linear actuator I obtained by kinetic simulation in SolidWorks software is 1,340 N. The theoretical calculation results and the kinetic simulation results have similar trends, which verifies that the kinetic modeling is accuracy of the dynamic modeling. The difference between the two results comes from the slight difference between the theoretical dynamic model and the actual mechanical structure. In this study, the linear actuator motor driving force is 1,500 N, and the self-locking force is 5,000 N. Therefore, the linear actuator can meet the requirements of use, i.e., the reconfigurable behavioral assistive robot can successfully assist the subject to perform the sit-to-stand transition.

## 3 Experiments and results

This study was approved by the Research Ethics Committee of the Shenyang Institute of Automation. All subjects were given informed consent and an experienced technician attended each test. All experiments were conducted in the laboratory, and before each experiment, subjects were asked to familiarize themselves with the exoskeleton and to adjust it accordingly.

### 3.1 Validation of the effectiveness of the prototype platform

To validate the effectiveness of the first-generation prototype platform of the reconfigurable behavioral assistive robot, we conducted no-load experiments on both the robot's assisted walking process and its conformational transformation process. These experiments are detailed below.

#### 3.1.1 Assisted walking process

**Figure 9:**
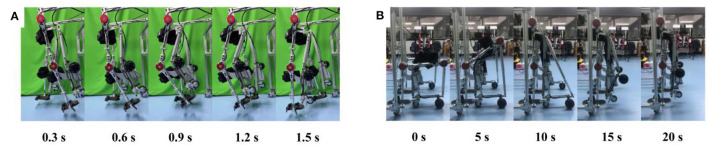
No-load experiment process. **(A)** Process of walking. **(B)** Process of conformal transformation.

In this experiment, we guided the robot through 30 complete gait cycles using a PD controller while it was in an unloaded state. The robot followed a pre-defined gait trajectory to assess the feasibility of the robot prototype during the walking process. The procedure for the walking experiment is illustrated in Figure 9A, where the robot is stationary at the 0 s moment. The results of the experiment indicate that the robot could more accurately trace the predetermined gait trajectory when it was in an unloaded state. This experiment served to demonstrate the effectiveness of the control system governing the exoskeleton state of the robotic device and affirmed its ability to assist subjects during walking.

#### 3.1.2 Conformal transformation process

In this experiment, we executed the process of conformal transformation with the robot while it was in an unloaded state. The experimental procedure is depicted in Figure 9B, where the duration from 0 to 10 s represents the transition of the robot from a wheelchair state to a support state, and the period from 10 to 20 s signifies the transition from the support state to the exoskeleton state. The experimental results demonstrated that the reconfigurable behavioral assistive robot could successfully and efficiently execute configuration transitions. This confirmed the rationality of the mechanical structure of the device and established the feasibility of providing support for subjects to stand up and assisting subjects in transitioning from a sitting position to a standing one.

### 3.2 Wearable experiments for assisted walking

To assess the reconfigurable behavioral assistive robot's performance in aiding subjects with walking, we conducted two distinct experiments. In the initial experiment, subjects wore the reconfigurable behavioral assistive robot for assisted walking, and we tracked their gait trajectories utilizing the classical PD algorithm. The purpose was to validate the feasibility and stability of the robot's assistance during walking. In the second experiment, we performed walking trials both with and without the robot, measuring EMG signals from relevant muscles during the walking process. This aimed to determine whether the behavioral assistive robot could alleviate muscle fatigue during walking, thus serving as a walking aid.

A healthy subject without any leg disorders volunteered for the gait trajectory tracking experiment. To assess the effectiveness of trajectory tracking, we evaluated the experiment's results by calculating Pearson's correlation coefficient (*R*) between the reference trajectory and the actual tracked trajectory.

For the EMG signal experiment and plantar pressure experiment, six healthy subjects without leg disorders volunteered to participate. Each set of experiments was divided into experimental and control groups. The experimental group wore the behavioral assistive robot, while the control group did not. To mitigate the potential influence of muscle fatigue on the results, we conducted the experiments over 2 days for both groups, maintaining identical experimental procedures. The statistical analysis was carried out using SPSS software, employing the Wilcoxon rank-sum test (*p* < 0.05).

#### 3.2.1 Gait tracking analysis

Given that a majority of hemiplegic patients lack the ability to actively engage in walking, passive training becomes the predominant mode during the rehabilitation process for patients using lower limb exoskeleton robots. In cases where the user is entirely passive, position-based trajectory tracking control plays a pivotal role, as it enables the provision of continuous and repetitive exercise for the residual limb. Consequently, this section is dedicated to conducting gait trajectory tracking experiments using a variable configuration lower limb exoskeleton robot, employing the classical PD control algorithm.

**Figure 10:**
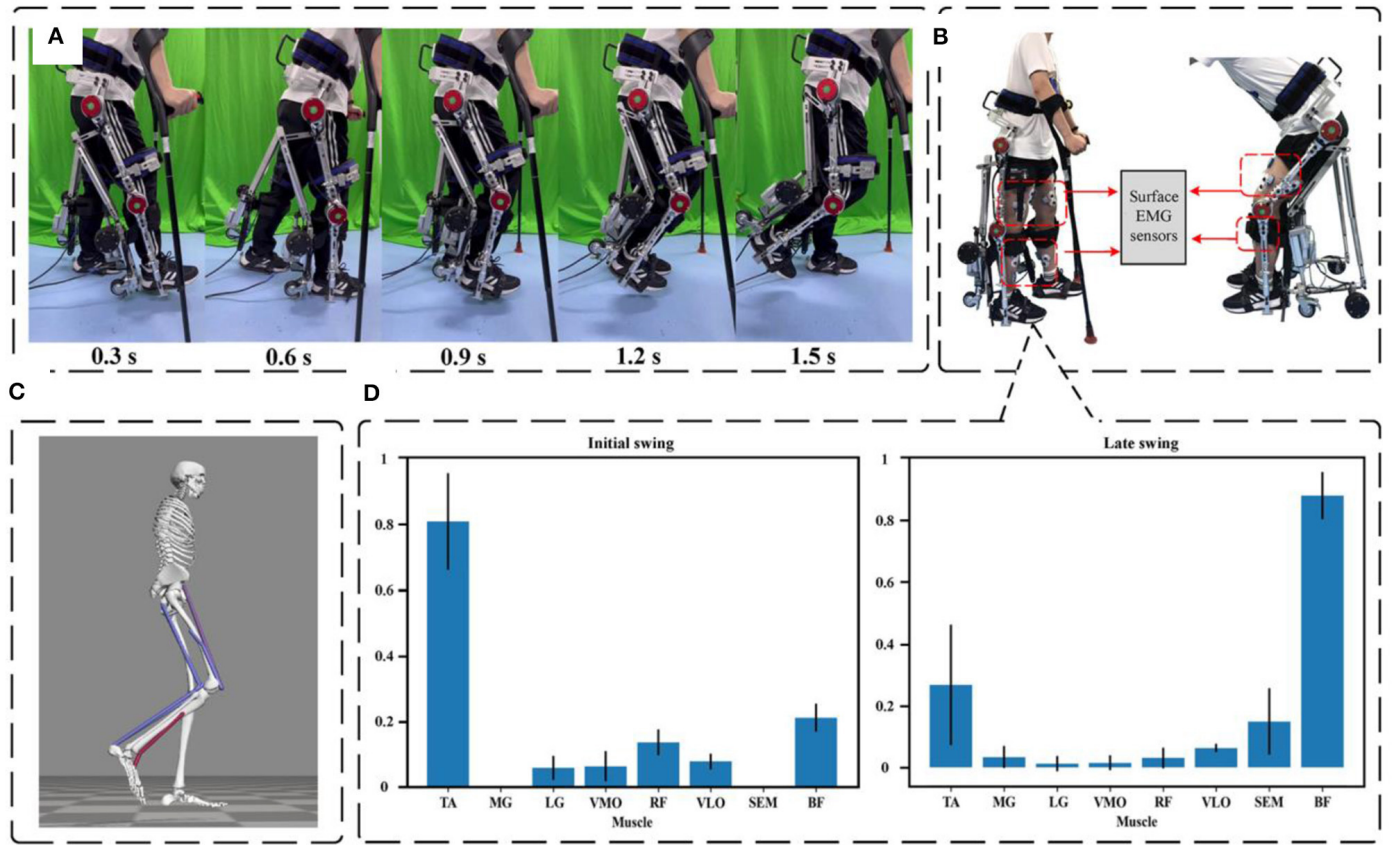
Experimental setup for assisted walking experiments. **(A)** Working process of robot-assisted walking. **(B)** Setup of the EMG experiments. **(C)** Opensim simulation process. **(D)** Degree of lower limb muscle contribution during the swing phase.

**Figure 11:**
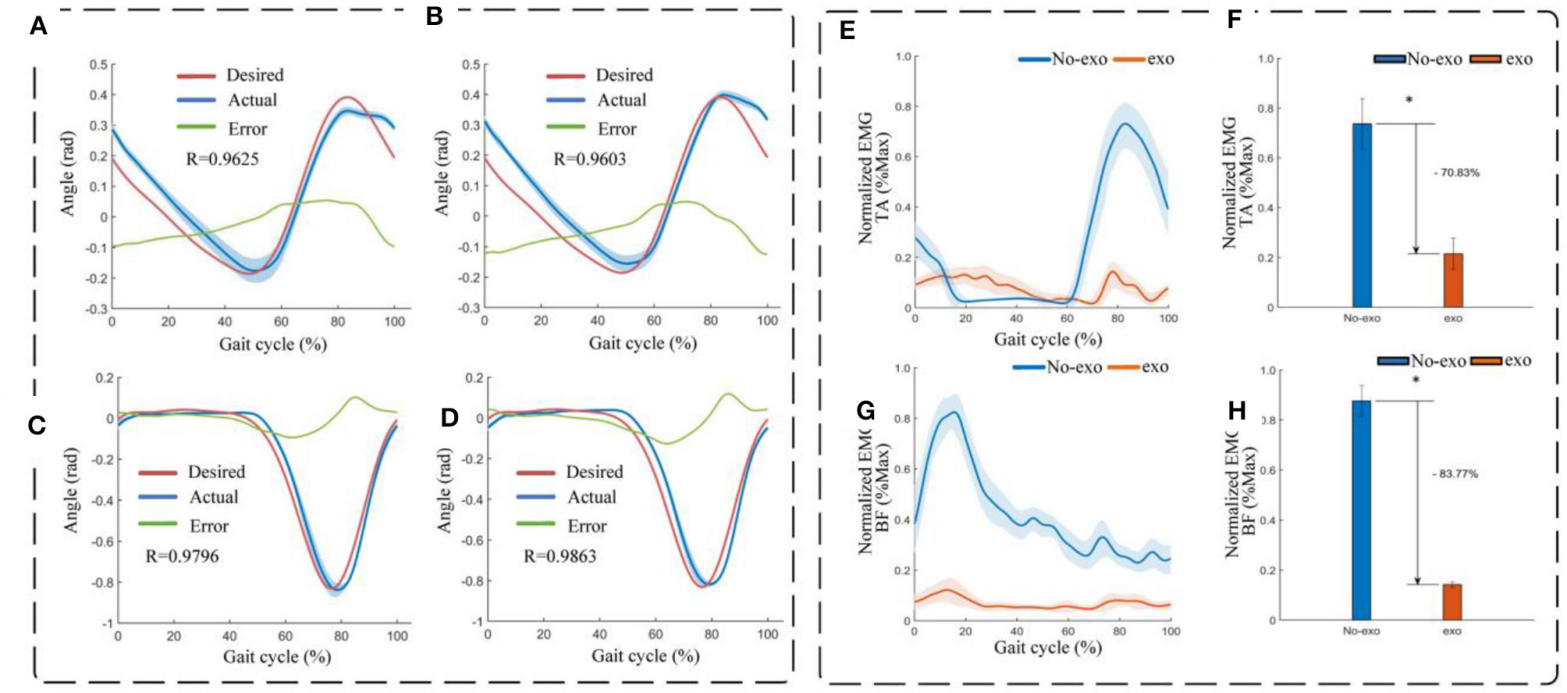
Experimental results of assisted walking experiments. **(A)** Left hip (flexion/extension) tracking results. **(B)** Right hip (flexion/extension) tracking results. **(C)** Left knee (extension/flexion) tracking results. **(D)** Right knee (extension/flexion) tracking results. **(E)** Envelope of EMG signals of the tibialis anterior (TA) in assisted walking experiments. **(F)** Normalized root-mean-square values of EMG signals of the tibialis anterior (TA) in assisted walking experiments. **(G)** Envelope of EMG signals of the biceps femoris (BF) in assisted walking experiments. **(H)** Normalized root-mean-square values of EMG signals of the biceps femoris (BF) in assisted walking experiments. Symbol * indicates statistically different results obtained before and after wearing the robot (Wilcoxon rank-sum test).

This experiment was conducted with a healthy participant who had a weight of 60 kg and a height of 175 cm. Once the participant wore the exoskeleton robot, control signals were transmitted from an industrial computer to the device. This allowed the exoskeleton robot to follow a predefined gait trajectory for tracking and walking, covering a span of 30 complete gait cycles. The predefined standardized gait trajectories were sourced from the CGA database (Reznick et al., 2021). Subsequently, joint angle values for both the right and left hip and knee joints were extracted during 15 of these gait cycles. The acquired data were subjected to a low-pass filter to obtain mean values, which were then normalized to a complete gait cycle. The experimental procedure is depicted in Figure 10A, where the robot is stationary at the 0 s moment. Figures 11A, B display the tracking results for the left and right hip joints, respectively, with errors falling within the (−0.1, 0.06) rad range. Figures 11C, D depict the tracking outcomes for the left and right knee joints. Notably, both the left and right knee joints exhibited errors within the (−0.1, 0.1) rad interval. To assess the trajectory tracking performance of the device under the PD controller, Figure 11 also provides the Pearson's correlation coefficient (*R*) between the reference trajectory and the actual trajectory, indicating the degree of correlation between them. Furthermore, an analysis of the tracking curves derived from the experiments reveals that the actual turning angles of both hip and knee joints on both sides of the exoskeleton closely align with the theoretical angles while achieving the desired gait postures. In other words, the subjects' actual gait postures closely resemble the standard postures of the robot during walking with the reconfigurable behavioral assistive robot. However, it's important to note that since the subject introduces perturbations to the PD controller during the tracking process, these perturbations are not actively mitigated by the controller. This phenomenon explains why trajectory tracking based on classical PD control may exhibit some phase lag as well as tracking errors. Overall, the experimental results indicate that the tracking performance of each joint angle in the reconfigurable behavioral assistive robot is generally satisfactory. This observation underscores that the robot, as designed in this study, possesses the capability to replicate various gait postures during the walking process and performs well in facilitating walking as well as passive rehabilitation training.

#### 3.2.2 Muscle activation evaluation

Many individuals suffering from walking disorders often experience deficits, such as insufficient forward thrust of the joints during the swing phase and inadequate foot clearance. These issues can lead to slow and uncoordinated gait patterns, ultimately resulting in instability and an increased risk of falling (Srivastava et al., 2015). Therefore, enhancing the smoothness and coordination of the swing phase in human walking is essential for restoring normal gait and reducing instability and the likelihood of falls. To investigate this, we utilized Opensim software to simulate the lower limb walking motion of humans. This simulation allowed us to assess the activation levels of the relevant muscles during the initial and final stages of the swing phase of human gait, as depicted in Figures 10C, D. In Figure 10D, the left part shows the degree of muscle contribution of the lower limbs at the initial swing and the right part shows the degree of muscle contribution at the late swing. The results of our simulation indicated that during the swing phase of gait, the primary muscles activated in the lower limbs were the tibialis anterior (TA) and biceps femoris (BF). Consequently, we selected these two muscles as the primary focus of our research to evaluate the effectiveness of the reconfigurable behavioral assistive robot in reducing muscle fatigue for patients during walking.

Surface electromyography (sEMG) signal analysis has been demonstrated as an effective method for assessing the electrical manifestations associated with localized muscle fatigue (Pi et al., 2006). To precisely evaluate the impact of the reconfigurable behavioral assistive robot on walking, we conducted an experiment in which we measured the EMG activity of muscles relevant to walking both with and without the presence of the robot. During the experiment, surface EMG signals were recorded from each subject at a sampling rate of 2 kHz using a wireless EMG system (Ultium EMG, Noraxon, USA). Specifically, we focused on monitoring the tibialis anterior (TA), lateral gastrocnemius (LG), hallux valgus (SOL), medial femoral (VM), rectus femoris (RF), lateral femoris (VL), semitendinosus (ST), and biceps femoris (BF) muscles. For each channel, two electrodes were positioned with a 20 mm spacing and affixed to the muscle belly of the subject's legs, aligning with the direction of the muscle fibers (Hermens et al., 2000; Chu et al., 2007; Satori et al., 2016; Zhang et al., 2021). These electrodes, composed of Ag-AgCl material, were connected to the EMG sensor through two wires. Subsequently, both the EMG sensor and the electrodes were securely attached to the subject's skin surface.

In the assisted walking experiment, participants were given specific instructions to perform two distinct gait maneuvers, one with the robotic assistance enabled and another without it. Initially, the device was connected and worn by each participant to initiate the experiment. An instruction was sent from the industrial computer to the device, activating it to provide assistance to the participant throughout 20 complete gait cycles. When the assistance was no longer needed, a signal was sent to the device to cease its operation, effectively discontinuing the walking assistance. Upon the conclusion of the trial in which all participants had utilized the behavioral assistive robot, the robot was removed from each participant. On the subsequent day, participants underwent another session in which they walked naturally for 20 complete gait cycles without wearing the robot. Prior to the start of each experiment, participants had undergone thorough practice sessions to ensure familiarity and proficiency. Figure 10B (left side) shows the experimental setup of the walking experiment.

The EMG data of the tibialis anterior (TA) and biceps femoris (BF) muscles before and after the subjects wore the robot were band-pass filtered (zero-lag fourth-order Butterworth, cutoff frequency 20–450 Hz), corrected, and low-pass filtered (zero-lag fourth-order Butterworth, cutoff frequency 6 Hz) in the software (MR 3.14, Noraxon, USA) The linear envelope was formed (Panizzolo et al., 2016). To facilitate comparisons, EMG amplitudes were normalized by calculating the average of peak EMG values observed during 10 consecutive stable gait cycles during walking. As illustrated in Figures 11E, F, the data revealed a significant reduction in muscle activation for both the tibialis anterior and lateral femoral muscles when the subjects engaged in assisted walking with the robot, compared to walking without the robot. During instances of muscle fatigue, the root-mean-square (RMS) of the EMG signal is considered a more suitable metric as it better reflects the muscle's contracted state (Jiang et al., 2017). The RMS values were computed for the filtered EMGs and then normalized to the maximum and minimum values. The results presented in Figures 11G, H demonstrate that with robot-assisted walking, the RMS of the EMG amplitudes for the tibialis anterior and lateral femoral muscles, both associated with the walking task, decreased significantly by 70.83% (*p* = 0.016) and 83.77% (*p* = 0.023), respectively. In conclusion, these findings suggest that the reconfigurable behavioral assistive robot effectively reduces muscle fatigue, yielding a substantial improvement in walking performance during assisted walking.

### 3.3 Wearable experiments for assisted standing-up

**Figure 12:**
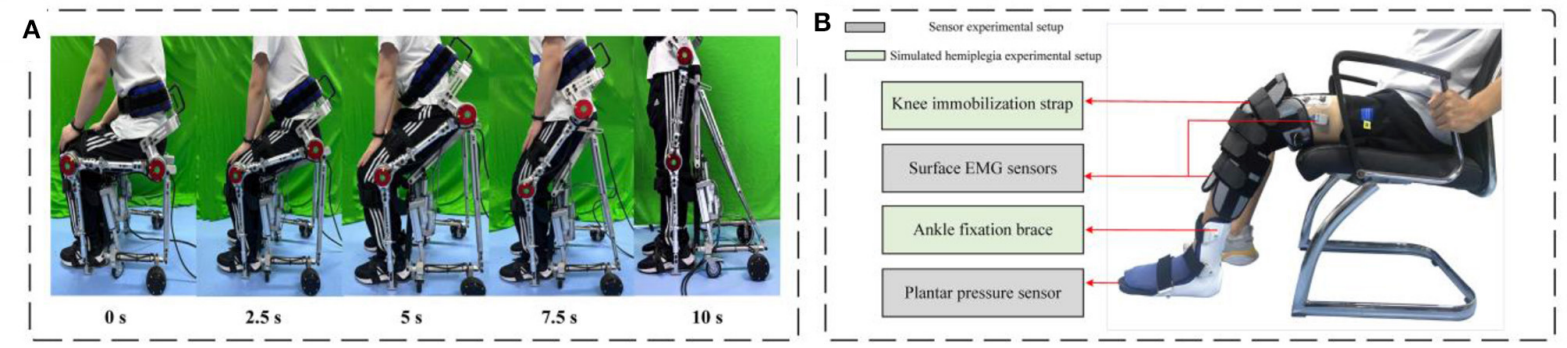
Experimental setup for assisted standing-up experiments. **(A)** Conformal transformation process of the robot during assisted standing up. **(B)** Experimental setup for assisted standing up experiment.

In order to evaluate the performance of a reconfigurable behavioral assistive robot in assisting subjects to stand, two different experiments were conducted. The first experiment was conducted on healthy subjects and measured their plantar pressure values and EMG signal values. This included the use of the robot and the non-use of the robot. This was done to assess the effective support provided by the behaviorally assisted robot during assisted standing as well as the reduced muscle fatigue. In the second experiment, we performed an assisted standing test facing a simulated hemiplegic patient and measured plantar pressure values and EMG signal values on the healthy and affected sides of the simulated hemiplegic patient, respectively. This included both with and without the presence of the robot. Six healthy subjects voluntarily participated in these experiments, and each experimental group was divided into both experimental and control groups, as described in Section 3.1. We conducted statistical analyses of the experiment results using the Wilcoxon rank-sum test (*p* < 0.05) with the assistance of SPSS software. The conformal transformation process of the assisted standing experiment is shown in Figure 12A.

#### 3.3.1 Experiments on healthy subjects

In experiments conducted on healthy subjects, the reconfigurable behavioral assistive robot aimed to alleviate muscle fatigue by redistributing the wearer's body weight, ensuring that it is not solely borne by the lower limbs but also transferred through the assistive robot and lower limbs combined. In order to assess the effectiveness of the robot in providing support during assisted rising and in reducing muscle fatigue, we measured the plantar pressures and EMG signals of subjects during rising with and without the robot. In this experiment, we first placed a fully instrumented wireless insole (Insole3, Moticon GmbH, Germany) in the subject's shoe and configured the subject with EMG sensors as described in Section 3.2.2. The device was then connected, and the subject, while seated in a wheelchair mode on the robot, commenced the trial. The plantar pressure insoles and EMG sensors are configured to collect data while subjects are assisted in standing up. During this period, the integrated control module sends a signal to the device to initiate the assisting actions. Once the subject is fully standing, the linear electric actuator of the robot automatically ceases operation. The entire process has a duration of 10 s. At the conclusion of the trial, the behavioral assistive robot was removed from the subjects. On the subsequent day, subjects engaged in a control experiment without wearing the robot. During this phase, subjects were instructed to sit on a chair adjusted to match the height of the robot's wheelchair state and to stand up from the chair at a constant speed, with the duration of this natural standing process equivalent to the duration of standing while wearing the robot, which lasted for 10 s. Each experiment was initiated after sufficient practice.

**Figure 13:**
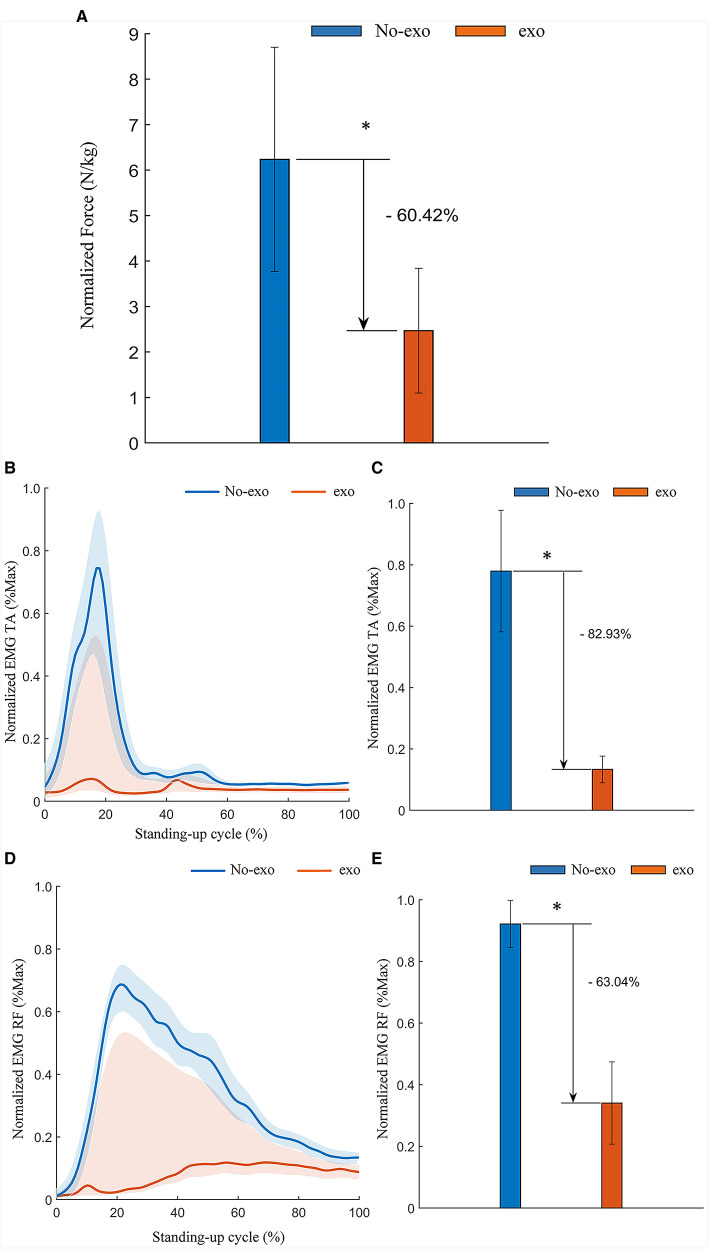
Experimental results of assisted standing-up experiments for healthy subjects. **(A)** Normalized root-mean-square values of plantar pressure in healthy subjects. **(B)** Envelope of EMG signals of the tibialis anterior (TA) in assisted standing-up experiments. **(C)** Normalized root-mean-square values of EMG signals of the tibialis anterior (TA) in assisted standing-up experiments. **(D)** Envelope of EMG signals of the rectus femoris (RF) in assisted standing-up experiments. **(E)** Normalized root-mean- square values of EMG signals of the rectus femoris (RF) in assisted standing-up experiments. Symbol * indicates statistically different results obtained before and after wearing the robot (Wilcoxon rank-sum test).

The plantar pressure data obtained from the insole were transmitted via Bluetooth to a mobile application (Moticon OpenGo, Moticon GmbH, Germany) for real-time monitoring and subsequently transferred to computer software (Moticon SCIENCE, Moticon GmbH, Germany) over a wireless network for storage and analysis. A standing cycle was defined as the duration from the commencement of the subject's standing movement to its completion. The pressure signal recorded from the wireless insole during one standing cycle served as the experimental result. To derive meaningful insights, the RMS was computed for the filtered plantar pressure values. To account for inter-subject variations, plantar pressures were normalized using each subject's respective body weight. The results revealed a significant 60.42% reduction in the RMS of plantar pressure (*p* = 0.028) when the behavioral assistive robot was utilized during assisted standing up, as depicted in Figure 13A. This evidence allows us to conclude that the reconfigurable behavioral assistive robot effectively supports the subject's body weight during assisted standing up, achieving a substantial booster effect.

Similarly, the EMG signals obtained from the EMG sensors during one standing cycle are the experimental data. The EMG signal data from the tibialis anterior (TA) and rectus femoris (RF) muscles, both before and after subjects wore the robot, were processed and filtered following the same procedures outlined in Section 3.1.2 to generate a linear envelope. The EMG amplitudes were then normalized using the average EMG signals recorded during the rise and fall exercises for each subject. As illustrated in Figures 13B, D, there was a significant decrease in muscle activation observed in the tibialis anterior and rectus femoris muscles. The RMS of the filtered EMG signals was computed and normalized against their respective maximum and minimum values. The results indicated a substantial reduction in the RMS of EMG amplitudes for both the tibialis anterior and rectus femoris muscles associated with the process of standing up. Specifically, there was an 82.93% reduction (*p* = 0.028) in the tibialis anterior muscle and a 63.04% reduction (*p* = 0.028) in the rectus femoris muscle when the behavioral assistive robot was employed to assist subjects during the standing-up process, as demonstrated in Figures 13C, E. In conclusion, the reconfigurable behavioral assistive robot effectively alleviated muscle fatigue in the relevant muscles, achieving a notable booster effect while assisting healthy subjects in the process of standing up.

#### 3.3.2 Experiments on simulated patient subjects

The primary target audience for reconfigurable behavioral assistive robots encompasses a significant population of hemiplegic patients, often resulting from strokes, as well as the elderly population facing mobility challenges. To further substantiate the behavioral assistive capabilities of the reconfigurable behavioral assistive robot, we employed healthy subjects to simulate hemiplegic patients. During the experimental process, we measured the plantar pressure values and EMG signal values of simulated patients transitioning from a seated position to a standing position, both with and without the assistance of the robot. These measurements were taken on the healthy side and the affected side of the simulated patients. In this way, we can assess the degree of effective support provided by the assistive robot during the process of helping hemiplegic patients stand, as well as the extent to which it alleviates muscle fatigue. Six healthy subjects voluntarily participated in this experiment. As described in Section 3.1, each experimental group consists of an experimental group and a control group. Statistical analysis of the experimental data was conducted using the Wilcoxon signed-rank test in SPSS software (*p* < 0.05).

Knee flexion defects are a common consequence of stroke, and they can result from a variety of contributing factors. One of these factors involves spasms caused by the overactivity of the rectus femoris muscle, while another key factor is the absence of adequate push-off at the ankle. The latter leads to a reduced rate of knee flexion when the toes are lifted off the ground, ultimately resulting in a deficiency of passive knee flexion (Goldberg et al., 2003; Campanini et al., 2013). To more accurately simulate the leg movement patterns observed in hemiplegic patients, we affixed medical knee immobilization straps and ankle immobilization supports to the legs of healthy subjects. This was done to mimic knee flexion defects attributable to rectus femoris muscle spasms and the insufficient ankle push-off forces characteristic of hemiplegic patients. The simulated leg morphology of these subjects is depicted in Figure 12B.

In the assisted sit-to-stand experiment designed for simulating hemiplegic patients, the configuration of the wireless insole and EMG system is identical to that employed in the experiment with healthy subjects. The experimental procedure is similar to the one described in Section 3.3.1 for healthy subjects. The subject is connected to the equipment and sits on the robot in a wheelchair mode to begin the experiment. Plantar pressure insoles and EMG sensors are configured to collect data as the subject is assisted in standing up. During this period, the integrated control module signals the device to initiate assisting actions After assisting the subject in fully standing up, the linear electric actuator of the robot stop operating. The entire process has a duration of 10 s. After the experiment concludes, the behavioral assistive robot is removed from the subject. The following day, the subject undergoes a control experiment without wearing the robot. At this stage, the subject is required to sit on a chair matched to the height of the robot in wheelchair mode and stand up from the chair at a uniform pace. The duration of this natural standing process matches the standing time when wearing the robot, lasting for 10 s. Each experimental set is conducted after thorough practice.

**Figure 14:**
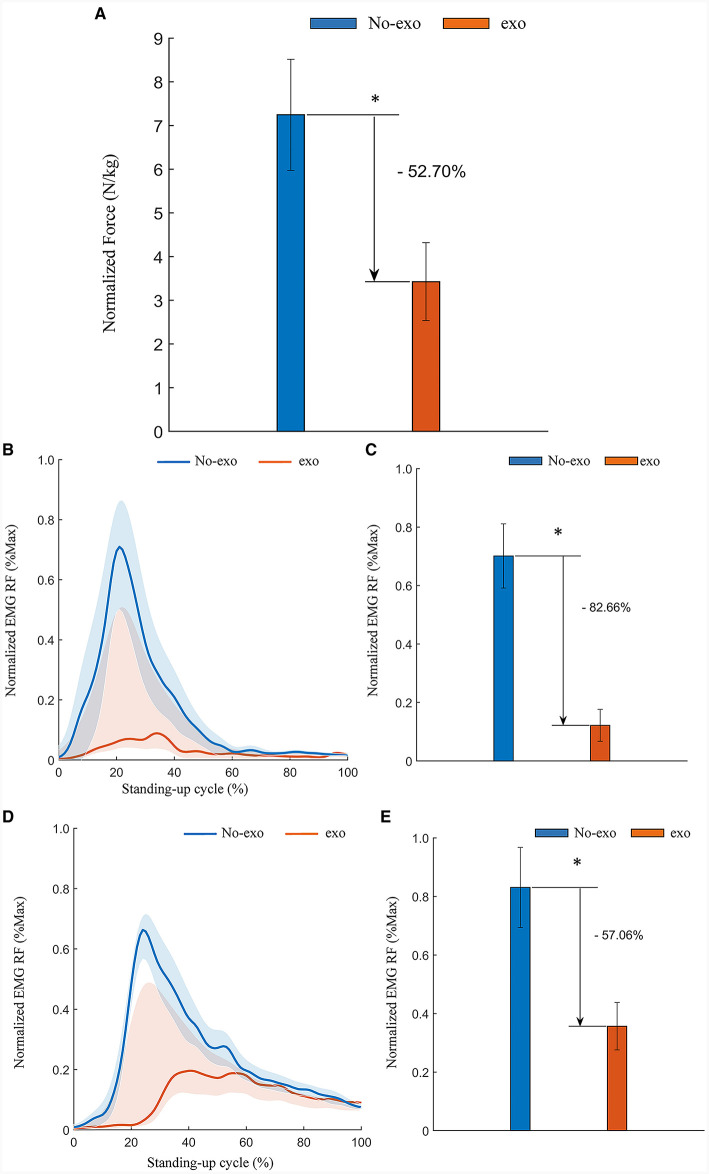
Experimental results of assisted standing-up experiments for simulated hemiplegic patients (healthy side). **(A)** Normalized root-mean-square values of plantar pressures on the healthy side of simulated hemiplegic patients. **(B)** Simulation of the tibialis anterior (TA) EMG signal envelope on the healthy side of a hemiplegic patient. **(C)** Normalized root-mean-square values of EMG signals of the tibialis anterior (TA) muscle on the healthy side of simulated hemiplegic patients. **(D)** Rectus femoris (RF) EMG signal envelope of the healthy side of a simulated hemiplegic patient. **(E)** Normalized root-mean-square values of EMG signals of the rectus femoris (RF) muscle on the healthy side of patients with simulated hemiplegia. Symbol * indicates statistically different results obtained before and after wearing the robot (Wilcoxon rank-sum test).

**Figure 15:**
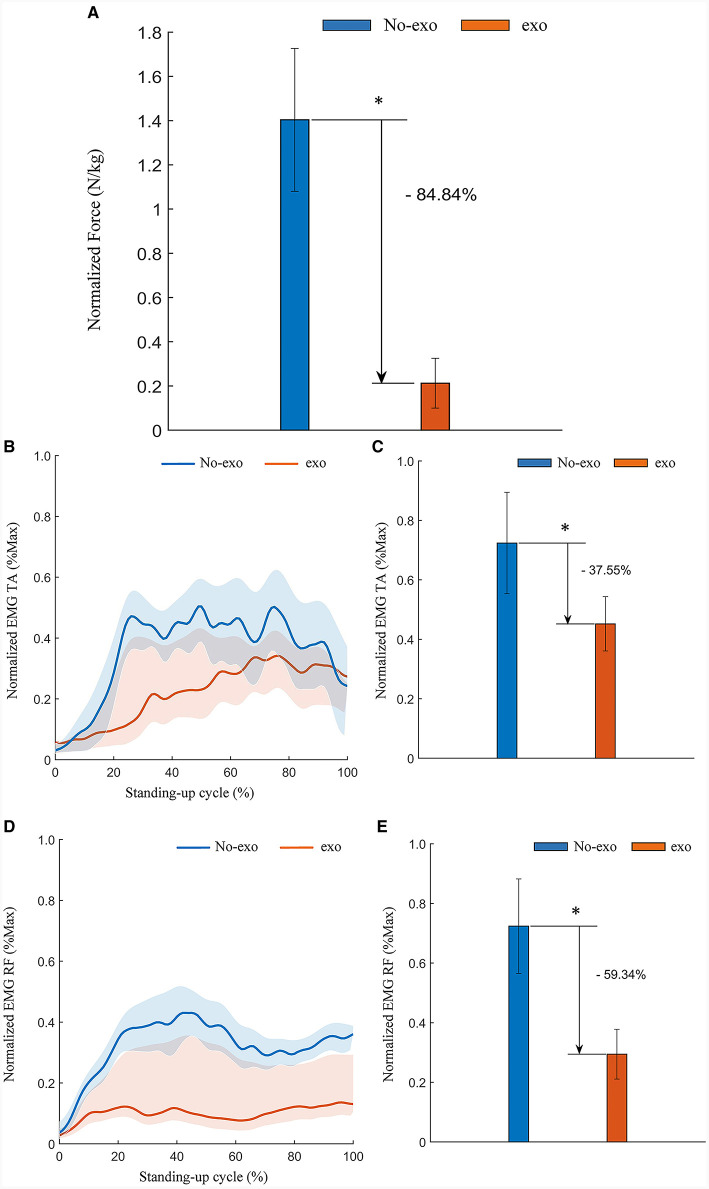
Experimental results of assisted standing-up experiments for simulated hemiplegic patients (affected side). **(A)** Normalized root-mean-square values of plantar pressures on the affected side of simulated hemiplegic patients. **(B)** Tibialis anterior (TA) EMG signal envelope of the affected side of a simulated hemiplegic patient. **(C)** Normalized root-mean-square values of EMG signals of the tibialis anterior (TA) muscle on the affected side of patients with simulated hemiplegia. **(D)** Rectus femoris (RF) EMG signal envelope of the affected side of a simulated hemiplegic patient. **(E)** Normalized root-mean-square values of EMG signals of the rectus femoris (RF) muscle on the affected side of patients with simulated hemiplegia. Symbol * indicates statistically different results obtained before and after wearing the robot (Wilcoxon rank-sum test).

The collected plantar pressure data on both the healthy and affected sides were processed and analyzed in a manner consistent with the approach described in Section 3.3.1. The results indicated a noteworthy reduction in the RMS of plantar pressure. Specifically, there was a 52.70% decrease (*p* = 0.028) on the healthy side and a substantial 84.84% decrease (*p* = 0.028) on the affected side of the simulated hemiplegic patients when the behavioral assistive robot was employed during assisted standing up. These findings are visually represented in Figures 14A, 15A. In summary, the reconfigurable behavioral assistive robot demonstrated its capability to effectively support the body weight of simulated hemiplegic patients during the process of assisted rising, thereby achieving a significant booster effect.

Similarly, data processing and analysis of EMG signals recorded from both the healthy and affected sides during the simulated hemiplegic patient experiment were conducted following the methods described in Section 3.1.2. As depicted in Figures 14B, D, there was a significant decrease in muscle activation observed in the tibialis anterior and rectus femoris muscles on the affected side of the simulated hemiplegic patients. The RMS of the filtered EMG signals was calculated and normalized to their respective maximum and minimum values. The results indicate that, with the assistance of the behavioral assistive robot during the process of standing up, the RMS of EMG amplitudes for the tibialis anterior and rectus femoris muscles on the affected side decreased by 37.55% (*p* = 0.046) and 59.34% (*p* = 0.028), as shown in Figures 14C, E respectively. Similarly, as illustrated in Figures 15B, D, there was a significant reduction in muscle activation in the tibialis anterior and rectus femoris muscles on the healthy side of the simulated hemiplegic patients after receiving robotic assistance. Again, the RMS of the filtered EMG signals was normalized. The results demonstrate that with the behavioral assistive robot assisting in the process of standing up, the RMS of EMG amplitude for the tibialis anterior and rectus femoris muscles on the healthy side decreased by 82.66% (*p* = 0.028) and 57.06% (*p* = 0.028), as shown in Figures 15C, E respectively. In conclusion, the reconfigurable behavioral assistive robot effectively reduces muscle fatigue in both the healthy and affected sides of the patient during the process of assisting the patient in standing up, resulting in a significant enhancement in performance.

## 4 Discussion

The above experiments were designed to validate the functionality of the designed reconfigurable behavioral assistive robot in achieving precise and consistent gait trajectory tracking. Furthermore, the study aimed to evaluate the robot's capacity to alleviate muscle fatigue in both healthy individuals and simulated hemiplegic patients during walking and standing up, while effectively providing support during their standing up motions. As expected, the reconfigurable behavioral assistive robot can accurately and consistently track joint angles. This capability suggests that the robot could assume an important role in assisting patients with their daily walking activities and passive rehabilitation training. In terms of muscle activation and plantar pressure, the reconfigurable behavioral assistive robot proves highly effective in assisting subjects during both walking and standing up, thus reducing muscle fatigue and providing valuable support. In experiments designed to simulate hemiplegic patients, it was observed that the affected side of the patient was unable to exert proper force due to impaired knee flexion, resulting in significantly lower muscle activation and plantar pressure on the affected side compared to the healthy side during the standing-up phase. Consequently, the patient's healthy side bore a greater support load and the potential for fatigue. The experimental findings highlight that the reconfigurable behavioral assistive robot not only serves as a valuable aid to the patient's affected side during the process of standing up but also alleviates support pressure and muscle fatigue on the patient's healthy side. It assumes a protective and supportive role for the patient's unaffected side.

The effectiveness of the reconfigurable behavioral assistive robot in assisting subjects to perform sit-to-stand transitions is verified at the level of theoretical analysis in kinetic modeling and dynamics simulation, where the main object of study and the index of measurement is the output force of the linear actuator motors, i.e., it is oriented to the “robot.” Furthermore, in a series of wearable experiments in Section 3, we verified at the experimental level the accuracy of the reconfigurable behavioral assistive robot's gait tracking and the effectiveness of assisting subjects in walking and standing-up, with the main measures being EMG signals and plantar pressures, i.e., “human” oriented. In summary, we have validated the effectiveness of a reconfigurable behavioral assistive robot in assisting subjects with walking and sit-to-stand transitions at both the “robot” and “human” levels.

### 4.1 Advantage of reconfigurable behavioral assistive robot

In this paper, we present the design of a reconfigurable behavioral assistive robot tailored to individuals with lower limb motor dysfunction. This innovative robot is capable of assisted walking, assisted standing up, supported standing, and provides wheeled mobility. Experiments were conducted to evaluate its effectiveness in assisting walking and standing. In previous studies, most multifunctional behavioral assistive robots faced disadvantages such as the need for disassembly, large size, and the inability to provide gait training after assisting users in standing (Hwang and Jeon, 2012; Asker and Assal, 2019; Li et al., 2019; Bell et al., 2021). Therefore, compared to previous research, this robot exhibits significant advantages: (1) It can seamlessly transition among the three configurations without disassembling, which is easier to operate and more intelligent. (2) It can assist walking and gait rehabilitation through the exoskeleton module after assisting the subject to move from sitting to standing. (3) The device is compact in size, and the configuration change process is safe and reliable, ensuring the balance and safety of the user. Therefore, the robot integrates the distinct advantages of an assisted standing wheelchair and a lower limb exoskeleton, effectively addressing a broader range of the subject's daily living requirements.

The distinctive feature of our reconfigurable behavioral assistive robot in terms of configuration design is the innovative adaptable deformation module set along the central sagittal plane of the legs. The thigh and calf support mechanisms are designed as swing-guided mechanisms in the supported state, while the thigh support mechanism transforms into a crank-slider mechanism in the exoskeleton state. The integration of the thigh support bars and thigh links with the interior of the exoskeleton module forms a crank-swing mechanism that promotes a seamless transition of the robot between wheelchair and exoskeleton modes. The proposed configuration offers several significant advantages over another common transformation configuration, the shear-fork mechanism (Zhang J. et al., 2022): (1) The number of required drives is less, while the ease of implementation and economy of the drive system is better than that of the shear-fork mechanism. (2) The proposed design requires fewer components, resulting in a reduced overall machine mass. (3) Only one limit pin is required in the proposed configuration to restrict the movement of the thigh support bar's moving vice, simplifying the mechanism switching process compared to the shear-fork mechanism.

In sum, this robot effectively combines the advantages of both a conventional wheelchair and a lower limb exoskeleton robot, skillfully circumventing the limitations inherent in each of these devices. Its exceptional versatility significantly enhances the user's motor capabilities, simultaneously fostering rehabilitative benefits for leg muscles. This innovative solution not only simplifies travel but also enhances the overall quality of life for individuals dealing with lower limb dysfunction. As a result, the development and implementation of this robot offer a promising avenue for enhancing the convenience and autonomy of the elderly and those with lower limb dysfunction, enabling them to lead more fulfilling lives, including the ability to venture out for travel. Such advancements can have a profound positive impact on their overall quality of life.

In addition, in this study, we choose to use EMG signals and plantar pressure as assessment indicators, as opposed to intent recognition and control mechanisms. The sEMG signals are widely recognized for their utility in monitoring muscle activity. In our work, the surface EMG signal module is capable of capturing signals via adhesive electrodes affixed to the specific muscles of interest. Additionally, we utilize a wireless pressure insole placed inside the subject's shoe to collect plantar pressure data. By measuring both electromyographic signals and plantar pressure throughout the behavioral assistive process, we objectively and comprehensively showcase the effectiveness and potential of the reconfigurable behavioral assistive robot in the realms of behavioral assistance and rehabilitation training. This approach provides a robust foundation for demonstrating the robot's capabilities and its impact on assisting individuals in need.

### 4.2 Study limitations and future work

The exoskeleton module of our reconfigurable behavioral assistive robot, as currently designed in this study, features a rigid structure. This design, while effective in certain aspects, lacks consideration for ground impact and does not incorporate cushioning or shock absorption capabilities. This oversight can affect the wearer's comfort during use. In our future work, we plan to address this limitation by incorporating shock absorption devices into the calf linkage of the robot. This enhancement will significantly contribute to enhancing the overall comfort and user experience. Another limitation worth noting is that the drive system of the conformal transformation module relies on a linear actuator motor, commonly found in rehabilitation equipment. This motor can only telescope at a constant speed and lacks the ability to adjust the speed and acceleration of the user's movement during the standing-up process. This limitation, while present in the current design, highlights an area for potential improvement in our future work. We aim to explore solutions that offer more flexibility and adaptability in controlling the user's movements, particularly during the critical phase of standing up.

The robot presented in this paper serves as the inaugural prototype of the reconfigurable behavioral assistive robot. It has successfully realized its intended functions, demonstrating commendable operational performance and effectiveness in assisting users. In forthcoming research endeavors, we plan to undertake several significant improvements and developments. Firstly, we intend to refine the robot's structural design, with an emphasis on creating an integrated drive unit characterized by high power density. This unit will be intricately linked to the existing robot framework established in this study. Additionally, we aspire to craft a body position adjustment system and introduce a versatile variable-configuration mobile support platform. Together, these enhancements will pave the way for the creation of a reconfigurable rehabilitation robot system capable of facilitating multi-stance rehabilitation training. At the level of control algorithms and human-robot interaction, our future work will encompass the incorporation of tailored control algorithms aimed at optimizing treatment prescriptions for patients with varying individual differences. In addition, the integration of voluntary participation and mechanical assistance in robot-assisted rehabilitation is crucial (Zhuang et al., 2021; Yang et al., 2023). Therefore, reconfigurable behavioral assistive robot systems are needed not only to enable motion-guided training of functionally impaired limbs for functional rehabilitation, but also for assistive synergy of patients' preserved locomotor abilities for on-demand assistance. Consequently, the collaboration and competition between patients and robots in human-robot interaction represent a significant challenge for the control system. To address this challenge, we plan to propose a human-robot-environment cohesive interaction control framework, which incorporates multi-level control research techniques, in which the low-level control performs the compliant interaction tasks at the joint level. The middle-level control accurately and robustly realizes the gait phase recognition, behavioral state feedback and perception of the human-robot-environment cohesive system during the motion process by means of the finite state machine on the basis of the low-level control, and thus change the control gain of the low level. The high-level controller consists of intent and event estimators, and physiological representations such as EMG and EEG are also introduced to issue control commands to the middle layer control.

## 5 Conclusion

In summary, we have developed a reconfigurable behavioral assistive robot capable of performing various functions, including transporting the user, aiding in sitting up, providing support for standing, and facilitating walking through the adaptation and reconfiguration of its mechanism. The first-generation prototype of this reconfigurable behavioral assistive robot has been successfully assembled by integrating multiple modules under a unified control system. It has demonstrated promising results in tasks such as unloaded walking, configuration adjustments, assisted walking, and aiding in standing up. In the assisted walking experiments, the robot exhibited precise tracking of the subjects' gait. Additionally, a notable reduction in the primary force-generating muscles in the lower limbs during walking was observed in subjects wearing the robot for assisted walking, in contrast to those not utilizing the robot. In the assisted rising experiments, a noteworthy reduction in the activation of the primary force-generating muscles in the lower limbs was observed in both healthy subjects and simulated hemiplegic patients during the rising process when they used the reconfigurable behavioral assistive robot. Furthermore, the subjects exhibited a significant decrease in plantar pressure values. These findings underscore the robot's capability to offer effective assistance and support to users during this crucial activity. Future research endeavors will focus on addressing existing limitations and implementing tailored control algorithms to optimize the robot's assistance for individualized patient needs.

## Data availability statement

The raw data supporting the conclusions of this article will be made available by the authors, without undue reservation.

## Ethics statement

The studies involving humans were approved by Shenyang Institute of Automation's Research Ethics Committee. The studies were conducted in accordance with the local legislation and institutional requirements. The participants provided their written informed consent to participate in this study. The animal study was approved by Shenyang Institute of Automation's Research Ethics Committee. The study was conducted in accordance with the local legislation and institutional requirements. Written informed consent was obtained from the individual(s) for the publication of any potentially identifiable images or data included in this article.

## Author contributions

ES: Conceptualization, Data curation, Formal analysis, Methodology, Validation, Writing – original draft. WZ: Formal analysis, Software, Validation, Writing – original draft. WC: Conceptualization, Methodology, Validation, Writing – original draft. YH: Conceptualization, Formal analysis, Methodology, Writing – original draft. BZ: Investigation, Project administration, Resources, Supervision, Writing – review & editing. XZ: Funding acquisition, Project administration, Resources, Supervision, Writing – review & editing.
